# The Effects of Antipsychotic Dose Reduction on Movement Disorders and Cardiometabolic Indices in Patients Remitted from a First Episode of Psychosis

**DOI:** 10.1093/schbul/sbaf116

**Published:** 2025-08-21

**Authors:** Yinzhao Liu, Iris E Sommer, Marieke J H Begemann, Shiral S Gangadin, Benjamin I Perry, Toon A W Scheurink, Nico J M van Beveren, HAMLETT and OPHELIA Consortium, Iris Sommer, Lieuwe de Haan, Wim Veling, Jim van Os, Filip Smit, Marieke Begemann, Sanne Koops, Machteld Marcelis, Martijn Kikkert, Nico van Beveren, Nynke Boonstra, Bram-Sieben Rosema, Roberto Bakker, Sinan Gülöksüz, Joran Lokkerbol, Ben Wijnen, Bodyl Brand, Shiral Gangadin, Erna van’t Hag, Priscilla Oomen, Alban Voppel, Franciska de Beer, Sterre Kamphuis, Iris Hamers, Matej Djordjevic, Toon Scheurink, Jort Noorman, Therese van Amelsvoort, Maarten Bak, Steven Berendsen, Truus van den Brink, Gunnar Faber, Koen Grootens, Martin de Jonge, Henderikus Knegtering, Jörg Kurkamp, Gerdina Hendrika Maria Pijnenborg, Anton Staring, Natalie Veen, Selene Veerman, Sybren Wiersma, Albert Batalla, Ruben Curfs, Jan-Jaap Hage, Ellen Graveland, Joelle Hoornaar, Inge Hobus, Karin Huizer, Pieter Roberto Bakker, Sanne Koops

**Affiliations:** Department of Biomedical Sciences, Cognitive Neuroscience Center, University Medical Center Groningen (UMCG), University of Groningen, Groningen 9713 GZ, The Netherlands; Department of Biomedical Sciences, Cognitive Neuroscience Center, University Medical Center Groningen (UMCG), University of Groningen, Groningen 9713 GZ, The Netherlands; Department of Biomedical Sciences, Cognitive Neuroscience Center, University Medical Center Groningen (UMCG), University of Groningen, Groningen 9713 GZ, The Netherlands; Department of Biomedical Sciences, Cognitive Neuroscience Center, University Medical Center Groningen (UMCG), University of Groningen, Groningen 9713 GZ, The Netherlands; Institute for Mental Health, University of Birmingham, Birmingham B15 2TT, United Kingdom; Department of Biomedical Sciences, Cognitive Neuroscience Center, University Medical Center Groningen (UMCG), University of Groningen, Groningen 9713 GZ, The Netherlands; Department of Biomedical Sciences, Cognitive Neuroscience Center, University Medical Center Groningen (UMCG), University of Groningen, Groningen 9713 GZ, The Netherlands; Parnassia Group for Mental Health Care, The Hague 2552 KS, The Netherlands; Department of Neuroscience, Erasmus Medical Centre, Rotterdam 3015 GD, The Netherlands; Department of Psychiatry, Erasmus Medical Centre, Rotterdam 3015 GD, The Netherlands; Department of Biomedical Sciences, Cognitive Neuroscience Center, University Medical Center Groningen (UMCG), University of Groningen, Groningen 9713 GZ, The Netherlands; Arkin, Institute for Mental Health, Amsterdam 1033 NN, The Netherlands; Department of Psychiatry and Neuropsychology, School for Mental Health and Neuroscience (MheNS), Maastricht University Medical Centre, Maastricht 6229 ER, The Netherlands; Department of Biomedical Sciences, Cognitive Neuroscience Center, University Medical Center Groningen (UMCG), University of Groningen, Groningen 9713 GZ, The Netherlands

**Keywords:** antipsychotic agents, dose dependency, metabolic disturbances, extrapyramidal side effects

## Abstract

**Background and Hypothesis:**

The extent to which tapering antipsychotic (AP) attenuates AP-related movement disorders and cardiometabolic dysfunction remains unclear. We aim to investigate the long-term effects of AP-dose reduction on these adverse effects in patients remitted from a first episode of psychosis (FEP).

**Study Methods:**

We included 293 FEP participants from the HAMLETT trial. Movement disorders were assessed using the St. Hans Rating Scale (SHRS) and Barnes Akathisia Rating Scale. Cardiometabolic indices included body mass index (BMI), waist circumference, blood pressure (BP), glucose, triglycerides, and cholesterol. Linear mixed-effects models assessed longitudinal relationships between AP-dose reduction, movement disorders and cardiometabolic indices.

**Study Results:**

Over an average 29-month follow-up (SD = 19), a 1 mg olanzapine equivalent dose reduction from baseline was associated with a 0.013-point decrease in Parkinsonism (95% CI, −0.019, −0.006), a potential 0.003-point decrease in tardive dyskinesia (95% CI, −0.006, −0.000) on SHRS (range 0-6), and decreases of 0.037 (0.15%) kg/m^2^ in BMI (95% CI, −0.059, −0.015), 0.153 (0.17%) cm in waist circumference (95% CI, −0.265, −0.037), 0.023 (0.47%) mmol/L in total cholesterol (95% CI, −0.039, −0.007), 0.018 (0.60%) mmol/L in low-density lipoprotein cholesterol (95% CI, −0.032, −0.003), and 0.021 (0.58%) mmol/L in nonhigh-density lipoprotein cholesterol (95% CI, −0.037, −0.005). We found no evidence for an association with tardive dystonia, akathisia, BP, glucose, or triglycerides.

**Conclusions:**

AP-dose reduction modestly benefits AP-related Parkinsonism, weight gain, cholesterol levels and potentially tardive dyskinesia in patients after FEP over time. These benefits should be carefully weighed against the risks of relapse and suicide.

## Introduction

Antipsychotic (AP) drugs are widely used to treat schizophrenia spectrum and other psychotic disorders and are often continued after remission to prevent relapse.^1^ However, AP often cause debilitating adverse effects. Movement disorders and cardiometabolic dysfunction are 2 of the most common adverse effects of AP.^2^ In AP users, the overall prevalence of movement disorders is 37%, and the prevalence of Parkinsonism, akathisia, and tardive dyskinesia is 20%, 11%, and 7%, respectively.^3^ Reported prevalences of metabolic syndrome among AP users range from 37% to 63%.^4^ The severity of movement disorders is longitudinally linked to individuals’ social functioning over time,^5^ and cardiometabolic dysfunction contributes to increased cardiovascular death rate.^6^ Moreover, the experience of AP-related movement disorders and weight gain can lead to stigma and treatment nonadherence.^7^^,^^8^

Recent meta-analyses link higher AP doses to increased risks of movement disorders and metabolic syndrome,^9–12^ but findings are scarce and contradictory. To protect patients against long-term adverse effects, many clinicians reduce AP dose after remission. However, it is still unclear to what extent a dose reduction can alleviate adverse effects. A recent systematic review of randomized controlled trials (RCTs) reported that AP-dose reduction compared to dose-maintenance was: (1) related to a small decrease in overall movement disorders scale score; (2) not associated with changes in the scores of each movement disorder scale; and (3) related to fewer patients with clinically significant weight gain (≥7% increase in weight from baseline), but not to actual weight reduction. The insignificant findings may be partly attributable to the low number of studies included in the systematic review, ranging from 3 to 9, and these studies often reported contradictory findings for both movement disorders and weight outcomes. Importantly, the follow-up periods of the included studies were mostly 1 year or less, limiting their ability to reflect the long-term effects of AP-dose reduction on movement disorders and weight. Additionally, the effect of AP-dose reduction on other cardiometabolic indices such as waist circumference, blood pressure (BP), and metabolic measures in blood was not summarized.^13^

Recently, the Research into Antipsychotic Discontinuation and Reduction (RADAR) trial in 253 patients with multiple episodes of psychosis reported that AP-dose tapering, compared to maintenance, was not related to changes in adverse effects as measured by the Glasgow Antipsychotic Side-effect Scale (GASS, including movement disorder measurements), nor in body weight. This large study with a follow-up of 24 months suggests that AP-dose reduction may not mitigate weight gain or movement disorders in patients with multiple episodes of psychosis. In the RADAR trial, 93% of participants had been in contact with mental health services for over 3 years.^14^ Less is known about the effects of AP-dose reduction on movement disorders and cardiometabolic dysfunction in the early onset of the disorder.

This study aims to investigate the long-term associations between AP-dose reduction and changes in movement disorders and cardiometabolic indices in a relatively large group of people who are in the early phase of treatment, being in remission from a first episode of psychosis (FEP) for 3 to 6 months.

## Methods

### Participants and Ethics

The data used in this study originates from the ongoing RCT Handling Antipsychotic Medication Long-term Evaluation of Targeted Treatment (HAMLETT). In the HAMLETT study, patients who had experienced a first episode of schizophrenia, schizoaffective disorder, schizophreniform disorder, brief psychotic disorder, delusional disorder, substance/medication-induced psychotic disorder, or those classified as Unspecified Schizophrenia Spectrum and Other Psychotic Disorders (according to the Diagnostic and Statistical Manual of Mental Disorders—5, or the International Classification of Diseases—10), and were in remission for 3-6 months, were randomized 1:1 to either AP continuation or early tapering/discontinuation. Written informed consent was obtained from all patients. Detailed information on the HAMLETT study is provided by Begemann et al.^15^ Data were collected at baseline, after 3 months, after 6 months, and then annually for 10 years. This study included participants recruited from the study’s inception in 2017 to May 2023, for whom baseline data on AP use were available. At the time of data extraction for the current analyses, follow-up information was available until January 2024. Participants with fewer than 2 observations with available AP-dose information were excluded. Selection bias was assessed by comparing characteristics of included and excluded participants.

The authors assert that all procedures contributing to this work comply with the ethical standards of the relevant national and institutional committees on human experimentation and with the Helsinki Declaration of 1975, as revised in 2013. All procedures involving patients were approved by the ethics committee of the University Medical Center of Groningen (METC number 2017-343).

### Variables and Measures

The AP dose was standardized to the olanzapine equivalent dose by combining the 95% effective dose, defined daily dose, and minimum effective dose methods.^16–18^ We primarily used the 95% effective dose because it is considered the most robust method to date.^18^ When data on the 95% effective dose were unavailable, we used the minimum effective dose instead. The defined daily dose method was used only as a last resort, as it is less evidence-based.^17^ Hereafter, this standardized value is referred to as “*AP dose*” throughout the text. Given that AP drugs vary in their movement disorder risks based on Dopamine-D2 receptor (D2R) affinity, following Kaar et al., AP drugs were categorized into low-medium D2R affinity (Ki > 10), high D2R affinity (Ki < 10), and partial D2R agonist using the National Institute of Mental Health’s Psychoactive Drug Screening Program database.^19^^,^^20^ Additionally, following Perry et al., we classified AP drugs as being more or less metabolically active (binary), depending on whether they have higher or lower metabolic risk.^21^ Patients who switched AP drugs between different D2R affinity or metabolic risk groups during follow-up were also captured (Table S1).

Antipsychotic-dose reduction was calculated from baseline to each visit (hereafter referred to as “AP-dose reduction from baseline”), determined by subtracting the dose at each visit from the baseline dose. To facilitate interpretation, dose reduction values were recentered by multiplying by −1.

Parkinsonism, tardive dyskinesia, and tardive dystonia were assessed using St. Hans Rating Scale (SHRS), while akathisia was measured using the Barnes Akathisia Rating Scale (BARS), all conducted by trained researchers. The SHRS includes 8 items for Parkinsonism and tardive dyskinesia, and a global score for tardive dystonia, all ranging from 0 (“absent”) to 6 (“severe”). Barnes Akathisia Rating Scale rates akathisia on one objective item and 2 subjective items from 0 (“absent”) to 3 (“severe”). We calculated the mean score of SHRS parkinsonism, tardive dyskinesia, and BARS akathisia per assessment to represent the degree of movement disorders, with lower or higher scores indicating less or more severe movement disorders, respectively. The validity and reliability of both scales are well-established.^22^^,^^23^ The SHRS and BARS scales were administered at baseline, 6 months post-baseline, and then yearly post-baseline during follow-up.

Cardiometabolic indices collected in the HAMLETT study include weight, height, waist circumference, BP, and metabolic indices in blood (glucose, triglycerides [TG], high-density lipoprotein cholesterol [HDL-C], non-high-density lipoprotein cholesterol [non-HDL-C], low-density lipoprotein cholesterol [LDL-C], and total cholesterol [TC]). Body mass index (BMI) was calculated by dividing weight by the square of height. Weight measures were available at baseline, after 3 months, after 6 months, and annually during follow-up. Cardiometabolic blood measures were obtained at baseline, and 6 months, 24 months and 48 months after baseline.

Movement disorder assessments and cardiometabolic indices were calculated as changes from baseline to each visit. Demographic variables including age, sex at birth, smoking status, diagnosis, substance use (amount of average weekly alcohol intake in the past month, frequency of hard drug use in the past month, and frequency of soft drug use in the past month) and medication information (AP type, daily dose, AP polypharmacy, other psychotropic medications, and medications for movement disorders and cardiometabolic dysfunction) were collected at each visit.

### Statistical Analyses

The data were analyzed in R (version 4.2.0) and RStudio (version 2022.07.02) for Windows, with the *lmerTest* (version 3.1-3) and *GLMMadaptive* (version 0.9-1) packages. The characteristics of included participants were described as mean ± standard deviation (SD) for continuous variables with an approximate normal distribution, median with interquartile range for skewed continuous variables, and numbers with percentages for categorical variables. We compared participants with missing weight and movement disorder data to those with complete data to assess potential bias (Tables S3–S5).

The relationship between AP-dose reduction and changes in movement disorders and cardiometabolic indices was evaluated using mixed-effect models with the change in AP-dose reduction from baseline to each visit as the independent variable, and the change in movement disorders and cardiometabolic indices from baseline to each visit as the dependent variables. Linearity between independent and dependent variables was examined using scatterplots and supported by prior evidence.^9–12^ Baseline observations were removed when modeling, as no dose reduction occurred at baseline.

Additionally, to provide more clinically relevant information, we followed the approaches of Pieters et al., Gerlach et al., and Barnes to apply cutoff values for defining cases of each movement disorder (detailed criteria are provided in the supplementary material, Chapter 4).^22–24^ We then used generalized mixed-effects models to examine the associations between AP-dose reduction and the odds of movement disorder cases over time.

To evaluate whether the effect of AP-dose reduction on movement disorders and cardiometabolic dysfunction depends on AP D2-affinity or metabolic risk groups, we introduced interaction terms between reduced AP olanzapine equivalent dose and D2R affinity, as well as between reduced AP dose and metabolic risk group, for the movement disorder and cardiometabolic indices analyses, respectively.

We also explored if preexisting movement disorders and cardiometabolic dysfunction at baseline affect the effect of AP-dose reduction on these adverse effects by: (1) introducing interaction terms between reduced AP olanzapine equivalent dose and preexisting movement disorders and cardiometabolic dysfunction; and (2) conducting subgroup analyses in patients with these preexisting conditions. See Supplementary material, Chapter 4, for detailed methods and results regarding cardiometabolic dysfunction (Tables S10 and S11).

Antipsychotic-dose reduction was also calculated and analyzed as time-lagged dose reduction. For those specific methods and results, please see Supplementary material, Chapter 7, Tables S15 and S16.

Moreover, we evaluated the associations between AP dose and movement disorders, as well as cardiometabolic indices. For those specific methods and results, please see Supplementary material, Chapter 8, Tables S17 and S18.

Sensitivity analyses were conducted for participants using only olanzapine to assess the stability of the overall AP results, as this AP type had the largest user group.

Analyses for both AP-dose reduction and AP dose were adjusted for baseline age, sex, smoking status, follow-up duration, AP switching during follow-up, concomitant use of other psychotropic medications (antidepressants, lithium, anticonvulsants, and benzodiazepines) and the baseline outcome values. As AP polypharmacy has been shown to be associated with a higher risk of movement disorders and cardiometabolic dysfunction,^25^^,^^26^ we also adjusted for AP polypharmacy as one of the covariates. For movement disorders, the models also included AP D2R affinity and concomitant use of medications that alleviate movement disorders (eg, biperiden) to adjust for their effects. The analyses of metabolic measurements were adjusted for the metabolic activity of AP (binary) and for the use of medications to mitigate metabolic syndrome, including lipid-lowering drugs, hypoglycemic agents, and antihypertensive drugs. Glucose analyses were adjusted for fasting status, as not all participants had fasted prior to glucose level measurements. We also included substance use variables as covariates to explore whether substance use influences AP-related movement disorders and cardiometabolic dysfunction. Detailed methods and results for these analyses are provided in the Supplementary material, Chapter 6, Tables S13 and S14. Results are presented as β coefficients with 95% confidence interval (CIs), representing the change in outcome units for each one-unit increase in the predictors.

## Results

### Participants

A total of 325 participants were initially considered, but 32 were excluded as accurate data on AP dose was missing. Consequently, 293 participants were included in the analyses. Table 1 and Table S2 show the demographic characteristics of the included participants. Of these, 68.9% were male. The mean age was 28.4 years (SD = 9.0). The largest proportion of participants was diagnosed with schizophrenia (46.1%). The mean AP dose at baseline was 9.1 mg (SD = 5.2). Most patients used olanzapine at baseline (38.9%), followed by aripiprazole (29%). The mean BMI at baseline was 25.1 kg/m^2^ (SD = 4.4), and the mean waist circumference was 92.3 cm (SD = 12.0). Most participants were free of tardive dyskinesia, akathisia, and tardive dystonia at baseline, as indicated by median scores of 0. Only SHRS mean scores for Parkinsonism showed a median of 0.4.

**Table 1:** Demographic and Clinical Characteristics of Included Participants

	**Participants (*n* = 293)**
Sex at birth (male), *n* (%)	202 (68.9%)
Age, mean (SD)	28.4 (9.0)
Follow-up durations (months), mean (SD)^a^	28.6 (18.5)
Smoking (yes), *n* (%)^b^	110 (39.4%)
*Diagnoses*	
Schizophrenia, *n* (%)	135 (46.1%)
Schizophreniform disorder, *n* (%)	73 (24.9%)
Schizoaffective disorder, *n* (%)	71 (24.2%)
Brief psychotic disorder, *n* (%)	13 (4.4%)
Other psychotic disorders, *n* (%)	1 (0.3%)
*Characteristics of AP use*	
Olanzapine equivalent dose, mean (SD)	9.1 (5.2)
Olanzapine, *n* (%)	114 (38.9%)
Dose of olanzapine users, mean (SD)	9.2 (4.4)
Aripiprazole, *n* (%)	85 (29.0%)
Olanzapine equivalent dose of aripiprazole users, mean (SD)	11.4 (5.9)
Others, *n* (%)	94 (32.1%)
Olanzapine equivalent dose of other AP users, mean (SD)	6.9 (4.6)
*AP D2R-affinity*	
Low–medium, *n* (%)	132 (45.1%)
High, *n* (%)	74 (25.3%)
Partial agonists, *n* (%)	87 (29.7%)
Metabolic risk (high), *n* (%)	169 (57.7%)
*Adverse effects*	
BMI, mean (SD)^c^	25.1 (4.4)
Waist circumference, mean (SD)^d^	92.3 (12.0)
Systolic BP, mean (SD)^e^	119.5 (14.0)
Diastolic BP, mean (SD)^e^	75.2 (9.5)
Parkinsonism SHRS mean scores (range 0-6), median (IQR)^f^	0.4 (0.5)
Dyskinesia SHRS mean scores (range 0-6), median (IQR)^g^	0 (0)
Dystonia SHRS global score (range 0-6), median (IQR)^h^	0 (0)
Akathisia SHRS mean scores (range 0-3), median (IQR)^i^	0 (0.7)

Abbreviations: AP, antipsychotic; SD, standard deviation; D2R, dopamine-D2 receptor; IQR, interquartile range.

a Based on data from 277 participants, as data for 16 participants were missing.

b Based on data from 279 participants, as data for 14 participants were missing.

c Based on data from 278 participants, as data for 13 participants were missing.

d Based on data from 237 participants, as data for 56 participants were missing.

e Based on data from 241 participants, as data for 52 participants were missing.

f Based on data from 254 participants, as data for 39 participants were missing.

g Based on data from 263 participants, as data for 30 participants were missing.

h Based on data from 268 participants, as data for 25 participants were missing.

i Based on data from 259 participants, as data for 34 participants were missing.

### Associations between AP-Dose Reduction and Changes in Movement Disorders and Cardiometabolic Indices Over Time

#### Movement Disorders

Over a mean follow-up period of 28.6 months (SD = 18.5), AP-dose reduction from baseline was related to a decrease in Parkinsonism (*β* = −0.013, 95% CI, −0.019, −0.006, *P* < .001). More specifically, 1 mg olanzapine equivalent of AP-dose reduction from baseline was associated with a decrease of Parkinsonism by 0.013 point on the SHRS (range 0-6). Additionally, we observed that AP-dose reduction from baseline was associated with a marginal decrease in tardive dyskinesia (*β* = −0.003, 95% CI, −0.006, −0.000, *P* = .052). More specifically, 1 mg of AP-dose reduction from baseline was associated with a potentially decrease of tardive dyskinesia by 0.003 point on the SHRS (range 0-6). We found no evidence for an association with akathisia or tardive dystonia. See Table 2 for the detailed results. The effect of each predictor on tardive dyskinesia is also shown in Figure S1.

**Table 2:** Effect of AP-Dose Reduction From Baseline Magnitude on Changes in Movement Disorder Measurements Over Time

**Outcomes**	**Variables**	**Estimates**	**95% CI**	***P*-value**
Parkinsonism^a^	Reduced dose	−0.013	−0.019, −0.006	<.001^***^
	Follow-up duration	0.001	−0.001, 0.003	.562
	*N* patients	207		
	*N* observations	473		
Tardive dyskinesia^a^	Reduced dose	−0.003	−0.006, −0.000	.052
	Follow-up duration	−0.001	−0.002, 0.001	.288
	*N* patients	215		
	*N* observations	493		
Akathisia^a^	Reduced dose	−0.000	−0.012, 0.003	.252
	Follow-up duration	0.001	−0.002, 0.004	.618
	*N* patients	210		
	*N* observations	483		
Tardive dystonia^a^	Reduced dose	−0.000	−0.001, 0.008	.983
	Follow-up duration	0.004	−0.000, 0.008	.050^*^
	*N* patients	223		
	*N* observations	525		

Abbreviations: AP, antipsychotic; CI, confidence interval.

^*^ *P* < .05.

^**^ *P* < .01.

^***^ *P* < .001.

a Adjusted for follow-up duration, age, sex, smoking status, AP D2R affinity groups, switch AP between different D2R affinity groups during follow-up, AP polypharmacy, concomitant use of other medications (antidepressants, benzodiazepines, mood stabilizers, anti-movement disorders medications) and baseline outcome values (baseline mean scores of Parkinsonism, dyskinesia, akathisia and global dystonia score, respectively).

For the defined cases of each movement disorder, we found that 1 mg reduction in olanzapine equivalent dose was associated with an 8.9% decrease in the odds of Parkinsonism (OR = 0.911, 95% CI, 0.872–0.953, *P* < .001) and a potential 6% decrease in the odds of tardive dyskinesia (OR = 0.940, 95% CI, 0.878–1.006, *P* = .074). No significant associations were observed for akathisia or dystonia. See Supplementary material, Chapter 5, Table S12 for detailed results.

Regarding the effect of AP-dose reduction in different D2R affinity groups, we found that, compared to low-to-medium D2R affinity AP, dose reduction of high D2R affinity AP was potentially more effective in decreasing mean Parkinsonism scores, but potentially associated with an increase in mean dyskinesia scores on the SHRS. We found no evidence that the effect of AP-dose reduction differed across D2R affinity groups for akathisia or dystonia. Please see detailed results in the Supplementary material, Chapter 3, Table S6.

We found that patients with preexisting Parkinsonism showed a greater effect of AP-dose reduction on decreasing parkinsonism mean scores (*β* = −0.016, 95% CI, −0.029, −0.004, *P* = .011), as well as dyskinesia mean scores (*β* = −0.014, 95% CI, −0.025, −0.003, *P* = .013), compared to those without Parkinsonism and dyskinesia at baseline, respectively. In contrast, patients with preexisting dystonia showed a greater increase in dystonia scores following AP-dose reduction (*β* = 0.031, 95% CI, 0.005, 0.057, *P* = .022) than those without preexisting dystonia at baseline. In subgroup analyses including only patients with preexisting movement disorders, we found that a 1 mg reduction in AP olanzapine equivalent dose was associated with a 0.017-point (95% CI, −0.026, −0.007, *P* = .001) decrease in Parkinsonism mean scores. No significant associations were found for tardive dyskinesia, akathisia, or tardive dystonia. Please see detailed results in the Supplementary material, Chapter 4, Tables S8 and S9.

#### Cardiometabolic Indices

Over a mean follow-up period of 28.6 months (SD = 18.5), AP-dose reduction from baseline was associated with a decrease in BMI (β = −0.037, 95% CI, −0.059, −0.015, *P* = .001), waist circumference (*β* = −0.153, 95% CI, −0.265, −0.037, *P* = .009), TC (*β* = −0.023, 95% CI, −0.039, −0.007, *P* = .007), LDL-C (*β* = −0.018, 95% CI, −0.032, −0.003, *P* = .022), and non-HDL-C (*β* = −0.021, 95% CI, −0.037, −0.005, *P* = .019), More specifically, a 1 mg reduction in olanzapine equivalents AP dose from baseline was associated with a BMI decrease of 0.037 (0.15%) kg/m and a waist circumference decrease of 0.153 (0.17%) cm. For blood indices, a 1 mg reduction in AP dose was associated with a decrease of TC, LDL-C, and non-HDL-C by respectively 0.023 (0.47%) mmol/L, 0.018 (0.60%) mmol/L, and 0.021 (0.58%) mmol/L. We found no evidence for an association with BP, glucose level, triglyceride, and HDL-C. See Table 3 for the detailed results.

**Table 3:** Effects of AP-Dose Reduction From Baseline Magnitude on Changes in Cardiometabolic Indices Over Time

**Outcomes** ^a^	**Variables**	**Estimates**	**95% CI**	***P*-value**
BMI^b^	Reduced dose	−0.037	−0.059, −0.015	.001^**^
	Follow-up duration	0.020	0.013, 0.027	<.001^***^
	*N* patients	260		
	*N* observations	863		
Waist circumference^b^	Reduced dose	−0.153	−265, −0.037	.009^**^
	Follow-up duration	0.070	0.037, 0.104	<.001^***^
	*N* patients	200		
	*N* observations	494		
Systolic BP^c^	Reduced dose	0.137	−0.041, 0.311	.133
	Follow-up duration	0.073	0.018, 0.129	.010*
	*N* patients	204		
	*N* observations	513		
Diastolic BP^c^	Reduced dose	0.081	−0.045, 0.204	.209
	Follow-up duration	0.092	0.054, 0.129	<.001^***^
	*N* patients	204		
	*N* observations	513		
Glucose^d^	Reduced dose	0.016	−0.017, 0.048	.381
	Follow-up duration	−0.023	−0.048, 0.003	.111
	*N* patients	86		
	*N* observations	103		
Triglycerides^e^	Reduced dose	−0.008	−0.026, 0.009	.360
	Follow-up duration	0.011	0.004, 0.019	.004^**^
	*N* patients	102		
	*N* observations	136		
Total Cholesterol^e^	Reduced dose	−0.023	−0.039, −0.007	.007^**^
	Follow-up duration	0.005	−0.001, 0.012	.126
	*N* patients	101		
	*N* observations	134		
HDL-Cholesterol^e^	Reduced dose	−0.003	−0.010, 0.004	.448
	Follow-up duration	−0.001	−0.004, 0.002	.461
	*N* patients	102		
	*N* observations	136		
LDL-Cholesterol^e^	Reduced dose	−0.018	−0.032, −0.003	.022^*^
	Follow-up duration	0.000	−0.006, 0.006	.967
	*N* patients	102		
	*N* observations	136		
non-HDL-Cholesterol^e^	Reduced dose	−0.021	−0.037, −0.005	.019^*^
	Follow-up duration	0.004	−0.002, 0.011	.233
	*N* patients	102		
	*N* observations	136		

Abbreviations: AP, antipsychotic; BP, blood pressure; BMI, body mass index; CI, confidence interval; HDL, high-density lipoprotein; LDL, low-density lipoprotein.

^*^ *P* < .05.

^**^ *P* < .01.

^***^ *P* < .001.

a For BMI, waist circumference, systolic BP, and diastolic BP, follow-up was conducted for up to 5 years. Glucose, triglycerides, TC, HDL-C, LDL-C, and non-HDL-C were followed up for up to 4 years.

b Adjusted for follow-up duration, age, sex, smoking status, AP metabolic risk group, switch AP between different metabolic risk groups during follow-up, AP polypharmacy, concomitant use of other medications (antidepressants, benzodiazepines, mood stabilizers, lowering glucose, lowering fat, antihypertensive drugs), and baseline outcome values (baseline BMI, waist circumference, respectively).

c Adjusted for follow-up duration, age, sex, smoking status, AP metabolic risk group, switch AP between different metabolic risk groups during follow-up, AP polypharmacy, baseline outcome values, concomitant use of other medications (antidepressants, benzodiazepines, mood stabilizers, lowering fat, antihypertensive drugs), baseline systolic and diastolic BP, respectively.

d Adjusted for follow-up duration, age, sex, smoking status, AP metabolic risk group, switch AP between different metabolic risk groups during follow-up, AP polypharmacy, baseline outcome values, fasting status, concomitant use of other medications, (antidepressants, benzodiazepines, mood stabilizers, antihypertensive drugs), and baseline glucose.

e Adjusted for follow-up duration, age, sex, smoking status, AP metabolic risk group, switch AP between different metabolic risk groups during follow-up, AP polypharmacy, concomitant use of other medications (antidepressants, benzodiazepines, mood stabilizers, antihypertensive drugs), baseline outcome values (baseline TG, TC, HDL-C, LDL-C, non-HDL-C, respectively).

Regarding the effect of AP-dose reduction in different metabolically active groups, we found that reducing the dose of more metabolically active AP was less effective in decreasing BMI, compared to reducing less metabolically active AP dose. We found no evidence that the effect of AP-dose reduction differed across metabolically active groups for other cardiometabolic indices, including waist circumference, systolic and diastolic BP, glucose, triglycerides, TC, LDL-C, HDL-C, and non-HDL-C. Please find detailed results in the supplementary material, Chapter 3, Table S7.

### Sensitivity Analyses: Including Only Olanzapine Users

The sensitivity analyses included 114 participants (68.4% males), with a mean follow-up period of 27.0 months (SD = 18.1). For movement disorders, we found an association between olanzapine-dose reduction from baseline and a decrease in tardive dyskinesia (*β* = −0.008, 95% CI, −0.013, −0.003, *P* = .002), meaning that a 1 mg olanzapine reduction from baseline was associated with a 0.008 decrease in tardive dyskinesia. We found no evidence for an association with Parkinsonism, akathisia or dystonia. For the cardiometabolic indices, we found that olanzapine-dose reduction from baseline was associated with a decrease in waist circumference (*β* = −0.219, 95% CI, −0.421, −0.007, *P* = .047), meaning that a 1 mg olanzapine-dose reduction from baseline was associated with a 0.219 cm decrease in waist circumference. We found no evidence for an association with the remaining cardiometabolic indices. Please see Supplementary material, Chapter 9, Tables S19–S24 for detailed results.

## Discussion

This study investigated the longitudinal association between AP-dose reduction, movement disorders and cardiometabolic indices in a large sample of patients with FEP. Over a mean follow-up period of 29 months, tapering AP was associated with significant, but modest decreases in Parkinsonism, BMI, waist circumference and cholesterol levels. We also found a marginally significant decrease in tardive dyskinesia. We found no evidence that AP-dose reduction was associated with tardive dystonia, akathisia, BP, glucose, or triglycerides in patients with FEP. In a sensitivity analysis including only 114 olanzapine users, only tardive dyskinesia and waist circumference remained significant, which may reflect a loss of power in this smaller subset. Notably, in terms of overall efficacy for psychotic symptoms, olanzapine has been suggested to be superior to several other AP, such as aripiprazole, risperidone, and quetiapine, in the treatment of acutely ill adults with psychosis.^27^ As a result, it remains one of the most commonly prescribed medications for the initial treatment of patients with FEP,^28^ and had the highest proportion among prescribed AP in our study. However, due to its high risk of cardiometabolic side effects, olanzapine is less favored as a first-line treatment for patients with FEP.^29^^,^^30^

This study provides evidence that AP-dose reduction in patients remitted from FEP is associated with decreased Parkinsonism, improved cardiometabolic indices and potentially decreased tardive dyskinesia, but these effects are modest. Previous studies on the effects of AP-dose reduction on movement disorders and cardiometabolic dysfunction are scarce and yielded contradictory results.^13^ Notably, our results differ from those of the RADAR trial,^14^ which did not find any benefits of AP-dose reduction in mitigating adverse effects. The present study included 293 patients after a FEP, with a mean follow-up period of 28.6 months (SD = 18.6), whereas the RADAR trial recruited 253 patients with chronic psychosis with a slightly shorter follow-up of 2 years. Patients in the early stages of treatment can be more prone to develop movement disorders and weight gain, and these effects tend to persist over time.^31–33^ Therefore, significant benefits of AP-dose reduction may be more evident in patients following a FEP and when assessed over a long-term follow-up. Power issues may also have been at stake, as retrieved effects were small, and our study was larger with a longer follow-up. Additionally, our approach of examining the actual AP-dose reductions from the baseline dose over time, instead of using intention-to-treat groups, may have allowed us to more precisely assess the association between AP-dose reduction and AP-related movement disorders and cardiometabolic indices over time.

Since the majority of our participants were not using high D2-affinity AP with limited prior exposure to AP, the prevalence and severity of movement disorders at baseline were low. Yet, we still observed associations between AP-dose reduction and Parkinsonism, as well as a potential association with tardive dyskinesia. Even small improvements in such adverse effects may provide meaningful benefits to patients.^34^

Interestingly, we found that reducing AP with low–medium D2R affinity may benefit tardive dyskinesia, whereas reducing high D2R affinity AP may worsen tardive dyskinesia. Although AP-dose reduction was generally suggested as a first-line management strategy for tardive dyskinesia in clinical practice,^35^ both the American Academy of Neurology guideline and Cochrane systematic review found little evidence that reducing AP-dose benefits tardive dyskinesia.^36^^,^^37^ Our findings provide important evidence that the effect of AP-dose reduction on alleviating tardive dyskinesia may depend on the D2R affinity of the AP. Consistently, Mentzel et al. also reported that starting a high D2 affinity AP may reduce the severity of tardive dyskinesia.^38^ This phenomenon also suggests that AP-dose reduction of high D2 affinity AP may require a slower and more cautious approach.^39^

The mechanisms underlying movement disorders are not fully understood, and several hypotheses have been proposed. The classical hypothesis states that Parkinsonism results from D2/3R blockade in the striatum, disrupting basal ganglia motor circuits. Studies show that over 80% striatal dopamine D2/3R occupancy is associated with Parkinsonism.^40^ Consistent with this hypothesis, tapering the AP dose is associated with lower dopamine receptor occupancy, which is linked to decreased Parkinsonism. Long-term dopamine receptor blockade may lead to receptor upregulation and dopamine hypersensitivity, which partly explains the development of tardive dyskinesia.^41^ Antipsychotics with lower D2R affinity, such as clozapine and quetiapine, have been suggested to dissociate rapidly from D2R and are therefore less likely to cause sustained receptor occupancy.^42^ This transient binding may reduce the risk of D2R upregulation and may explain the observed benefits of dose reduction in low D2R affinity AP. In contrast, high D2R affinity AP, such as haloperidol, have been shown to be associated with D2R upregulation in both human and animal studies. Tapering these medications could trigger exaggerated responses to endogenous dopamine, potentially worsening dyskinesia.^43^ Akathisia may primarily involve serotonin receptors, as similar symptoms can occur with selective serotonin reuptake inhibitors.^44^ Tardive dystonia is thought to result from adverse neuroplastic changes induced by D2R antagonists and anticholinergic medications, which disrupt the cortical-striatal-thalamic-cortical circuits. As these long-term changes are not directly related to dopamine receptor blockade, they may not be reversed through AP-dose tapering.^45^ This may explain why AP-dose reduction was not associated with akathisia or tardive dystonia in our analyses.

Although we found relatively small effect sizes, it is important to note that in clinical practice, AP doses are typically tapered by more than 1 mg olanzapine equivalents, which will lead to more remarkable benefits. For cardiometabolic indices, a 10 mg reduction in AP olanzapine equivalent dose would result in decreases of 1.53 (1.7%) cm in waist circumference, 0.23 (4.7%) mmol/L in TC, 0.18 (6%) mmol/L in LDL-C and 0.21 (5.8%) mmol/L non-HDL-C over time. Although still modest, even small improvements have a meaningful impact on cardiovascular risk over time. Solid evidence links waist circumference and LDL-C levels to cardiovascular diseases (CVD) and mortality.^46^^,^^47^ Waist circumference is recognized as an independent predictor of cardiovascular mortality apart from BMI. It is recommended as a key treatment target for lowering cardiometabolic risks by international guidelines.^46^ In addition, cumulative LDL-C exposure from young adulthood onwards is associated with an increased risk of cardiovascular events, highlighting the importance of early LDL-C control.^47^ Therefore, our findings provide important evidence that tapering AP doses in patients after FEP can reduce the risk of CVD and mortality. We did not observe a relationship between AP-dose reduction and changes in glucose levels, but this does not necessarily mean that tapering AP has no effect on glucose metabolism. Glucose homeostasis can be disrupted long before diabetes develops and may not be reflected in blood glucose levels. Also, schizophrenia spectrum disorders have been associated with disrupted glucose metabolism, independent of AP use.^48^ Therefore, while dose reduction may have improved glucose regulation, this improvement may not yet be detectable through glucose measurements.^49^

The effects of AP on dopamine, serotonin, acetylcholine, and histamine receptors in the hypothalamic arcuate and paraventricular nuclei play a central role in the mechanism of AP-related cardiometabolic adverse effects. By blocking these receptors, AP inhibits the expression of anorexigenic neuropeptides (eg, α-melanocyte-stimulating hormone), increases 5′AMP-activated protein kinase activity, and elevates sympathetic outflow. These effects collectively enhance appetite, promote glucagon secretion, and stimulate hepatic gluconeogenesis and glycogenolysis, subsequently disrupting glucose and lipid homeostasis as well as inducing weight gain.^50^ Tapering AP reduces the occupancy of the receptors and may thereby ameliorate AP-related cardiometabolic dysfunction.

Our findings underscore that tapering AP in patients after FEP may modestly reduce Parkinsonism, cardiometabolic indices, and potentially tardive dyskinesia, thereby possibly contributing to improved quality of life and reducing the risk of CVD and mortality. On the other hand, AP-dose reduction has been associated with an increased risk of relapse.^14^ Relapse and associated hospital admissions can be experienced as profoundly distressing and traumatic. Moreover, patients with a relapsing course have reduced chances of sustaining relationships, higher risks of unemployment and more severe functional and cognitive decline.^51^ Antipsychotics have been associated with lower suicidality and lower rates of suicide.^52^ Therefore, it is important to balance the potential benefits of reducing AP-related adverse effects with the increased risk of relapse and suicide when tapering AP doses.

### Limitations

This study has several limitations. Since our participants were not drug-naïve at baseline, no information was available on our main outcomes prior to AP treatment, nor on the AP trajectory before remission was achieved. The prevalence of spontaneous Parkinsonism and dyskinesia is 14% and 3%, respectively, in drug-naïve patients with FEP,^53^ and significant weight gain often occurs within the first 3 months of AP use.^31^ Additionally, clinicians may have adjusted the AP dose as soon as obvious adverse effects appeared, even before participants had reached remission, and were included in the HAMLETT study. These factors may lead to our results underestimating the effect of AP-dose reduction on movement disorders and cardiometabolic indices.^31^

The fact that different diagnostic groups were included should be taken into account. Some evidence suggests that patients with schizoaffective disorder have a 1.41-fold higher risk of metabolic syndrome compared to those with schizophrenia.^54^ However, patients with schizoaffective disorder are more likely to concomitantly use antidepressants, benzodiazepines, and mood stabilizers,^55^ which may contribute to the increased risk of AP-related adverse effects.^56^^,^^57^ As our sample consists of patients just after their FEP, their diagnoses may not yet be stable, limiting the meaningfulness of additional analyses based on diagnostic categories. Instead, we included the concomitant use of benzodiazepines, antidepressants, and mood stabilizers in our analyses to adjust for potential confounding effects from other psychotropic medications.

Some data were missing across various outcomes, which may have introduced bias into the estimates. Importantly, we did not perform multiple imputation prior to the mixed-effects model analysis, as these models use full information maximum likelihood procedures to estimate coefficients and standard errors, making multiple imputation unnecessary.^58^ An earlier study has shown that the results of mixed-model analyses with multiple imputation can be unstable.^59^ Furthermore, when we compared the baseline characteristics of participants with and without missing weight and movement disorder measurements, no differences were found in age, sex, smoking status, or olanzapine equivalent dose between the 2 groups. This suggests that the missing data is unlikely to introduce meaningful bias into our analysis.

Additionally, the glucose measurements were designed to reflect fasting levels. However, not all participants adhered to the fasting protocol before blood collection, which may have affected the accuracy of the fasting glucose data. To address this issue, we included fasting status as a covariate in the model.

## Conclusion

This study demonstrates that AP-dose reduction in FEP patients can decrease Parkinsonism, BMI, waist circumference, cholesterol levels and potentially also tardive dyskinesia. Although these reductions were modest, they may contribute to improved quality of life and meaningfully reduce cardiovascular risks and mortality, especially when dose reductions exceed 10 mg of olanzapine equivalent dose. Such dose reductions should be carefully balanced against the increased risk of relapse and suicide associated with tapering AP.

## Members of HAMLETT and OPHELIA Consortium

Iris Sommer^1^; Lieuwe de Haan^2^; Wim Veling^3^; Jim van Os^4–6^; Filip Smit^7–9^; Marieke Begemann^1^; Sanne Koops^1^; Machteld Marcelis^5,10^; Martijn Kikkert^11^; Nico van Beveren^12,13^; Nynke Boonstra^4,14,15^; Bram-Sieben Rosema^1^; Roberto Bakker^3,11^; Sinan Gülöksüz^5,16^; Joran Lokkerbol^9^; Ben Wijnen^9^; Bodyl Brand^38^; Shiral Gangadin^1,4^; Erna van’t Hag^3^; Priscilla Oomen^37^; Alban Voppel^1,17^; Franciska de Beer^1^; Sterre Kamphuis^5^; Iris Hamers^1^; Matej Djordjevic^3^; Toon Scheurink^1^; Jort Noorman^1^; Therese van Amelsvoort^5,18^; Maarten Bak^5,18^; Steven Berendsen^2,19^; Truus van den Brink^20^; Gunnar Faber^21^; Koen Grootens^22,23^; Martin de Jonge^24^; Henderikus Knegtering^3,25^; Jörg Kurkamp^26^; Gerdina Hendrika Maria Pijnenborg^27,28^; Anton Staring^29,36^; Natalie Veen^30^; Selene Veerman^31^; Sybren Wiersma^32^; Albert Batalla^4^; Ruben Curfs^33^; Jan-Jaap Hage^34^; Ellen Graveland^21^; Joelle Hoornaar^12^; Inge Hobus^1,35^; Karin Huizer^33,39,40^

^1^Department of Biomedical Sciences, University of Groningen, University Medical Center Groningen, Groningen, The Netherlands

^2^Department of Early Psychosis, Amsterdam UMC, Academic Medical Center, Amsterdam, The Netherlands

^3^Department of Psychiatry, University of Groningen, University Medical Center Groningen, Groningen, The Netherlands

^4^Department of Psychiatry, UMC Utrecht Brain Center, University Medical Center Utrecht, Utrecht, The Netherlands

^5^Department of Psychiatry and Neuropsychology, School for Mental Health and Neuroscience (MHeNs), Maastricht University Medical Centre, Maastricht, The Netherlands

^6^King’s College London, King’s Health Partners Department of Psychosis Studies; Institute of Psychiatry, Psychology & Neuroscience, London, United Kingdom

^7^Department of Epidemiology and Biostatistics, Amsterdam Public Health Research Institute, Amsterdam University Medical Centers, location VUmc, Amsterdam, The Netherlands

^8^Department of Clinical, Neuro and Developmental Psychology, Amsterdam Public Health Research Institute, Vrije Universiteit, Amsterdam, The Netherlands

^9^Centre of Economic Evaluation & Machine Learning, Trimbos Institute (Netherlands Institute of Mental Health), Utrecht, The Netherlands

^10^Institute for Mental Health Care Eindhoven (GGzE), Eindhoven, The Netherlands

^11^Department of Research, Arkin Mental Health Care, Amsterdam, The Netherlands

^12^Antes Center for Mental Health Care, Rotterdam, The Netherlands

^13^Department of Neuroscience, Erasmus MC, Rotterdam, The Netherlands

^14^NHL Stenden, University of Applied Sciences, Leeuwarden, The Netherlands

^15^KieN VIP Mental Health Care Services, Leeuwarden, The Netherlands

^16^Department of Psychiatry, Yale University School of Medicine, New Haven, Connecticut

^17^Douglas Mental Health University Center, McGill University, Montréal, Canada

^18^Mondriaan Mental Health Care, Heerlen, The Netherlands

^19^Dimence Institute for Mental Health, Deventer, Zwolle, The Netherlands

^20^Early Intervention Team, GGZ Centraal, Amersfoort, The Netherlands

^21^Yulius, Mental Health Institute, Dordrecht, The Netherlands

^22^Reinier van Arkel Institute for Mental Health Care, 's-Hertogenbosch, The Netherlands

^23^Tranzo, TSB, Tilburg University, Tilburg, The Netherlands

^24^Program for Psychosis & Severe Mental Illness, Pro Persona Mental Health, Wolfheze, The Netherlands

^25^Lentis Research, Lentis Psychiatric Institute, Groningen, The Netherlands

^26^Center for Youth with Psychosis, Mediant ABC Twente, Enschede, The Netherlands

^27^Department of Psychotic Disorders, GGZ-Drenthe, Assen, The Netherlands

^28^Department of clinical and developmental neuropsychology, faculty BSS, University of Groningen, Groningen, The Netherlands

^29^Department ABC Early Psychosis, Altrecht Psychiatric Institute, Utrecht, The Netherlands

^30^GGZ Delfland, Delfland Institute for Mental Health Care, Delft, The Netherlands

^31^Community Mental Health, Mental Health Service Noord-Holland Noord, Schagen, The Netherlands

^32^Early Intervention Psychosis Team, GGZ inGeest Specialized Mental Health Care, Hoofddorp, The Netherlands

^33^Parnassia Psychiatric Institute, The Hague, The Netherlands

^34^GGZ Breburg, Tilburg, The Netherlands

^35^Janssen-Cilag B.V., Breda, The Netherlands

^36^Psychologist Netherlands, Psychosis department, Utrecht, The Netherlands

^37^Behavioural Science Institute, Radboud University, Nijmegen, The Netherlands

^38^Department of Psychiatry, University of Oxford, Oxford, United Kingdom

^39^Department of Psychiatry, Amsterdam UMC, Academic Medical Center, Amsterdam, The Netherlands

^40^Department of Pathology, Erasmus MC, Rotterdam, The Netherlands

## Supplementary Material

supplementary_materials_SB_sbaf116
