# Supplementary material for: The Effects of Antipsychotic Dose Reduction on Movement Disorders and Cardiometabolic Indices in Patients Remitted from a First Episode of Psychosis
Source: Schizophr Bull. 2025 Aug 21;52(4):sbaf116. doi: 10.1093/schbul/sbaf116 (PMC13391666; doi:10.1093/schbul/sbaf116)
Supplement: supplementary_materials_SB_sbaf116 [file supplementary_materials_sb_sbaf116.docx]

**Chapters**

[**1.** **Characteristics of medication use and blood indices** 2](#_Toc198485230)

[**2.** **Comparisons between missing and non-missing data** 4](#_Toc198485231)

[**3.** **Effect of antipsychotic-dose reduction stratified by D2R affinity and metabolic risk** 8](#_Toc198485232)

[**4.** **Effect of antipsychotic-dose reduction in patients with preexisting movement disorders and cardiometabolic dysfunction at baseline** 14](#_Toc198485233)

[**5.** **Detailed results on the effect of antipsychotic-dose reduction from baseline magnitude on defined movement disorder cases** 26](#_Toc198485234)

[**6.** **Models adjusted for substance use** 27](#_Toc198485235)

[**7.** **Time-lagged antipsychotic-dose reduction analyses** 31](#_Toc198485236)

[**8.** **The association between antipsychotic dose and movement disorder and cardiometabolic indices** 36](#_Toc198485237)

[**9.** **Sensitivity analyses: including only olanzapine users** 42](#_Toc198485238)

[**10.** **Figure** 54](#_Toc198485239)

[**11.** **Reference** 55](#_Toc198485240)

# **Characteristics of medication use and blood indices**

Table S1. The characteristics of olanzapine equivalent dose, calculation method, dopamine receptor 2 affinity and metabolic risk of individual antipsychotics in the HAMLETT study.

| Antipsychotics | Olanzapine 1mg eq | Equivalent dose method | D2R affinity group | Metabolic risk group |
| --- | --- | --- | --- | --- |
| Aripiprazole | 0.76 | 95% effective doses | Partial agonist | No |
| Clozapine | 40 | Minimum effective dose | Low | Yes |
| Flupenthixol | 0.6 | DDD | High | No |
| Haloperidol | 0.42 | 95% effective doses | High | No |
| Olanzapine | 1 | - | Medium | Yes |
| Pimozide | 0.4 | DDD | High | Yes |
| Quetiapine | 31.78 | 95% effective doses | Low | Yes |
| Risperidone | 0.41 | 95% effective doses | High | Yes |
| Zuclopentixol | 3 | DDD | High | No |
| Sulpiride | 80 | DDD | High | No |
| Penfluridol | 0.6 | DDD | High | No |
| Paliperidone | 0.88 | 95% effective doses | High | Yes |
| Amisulpride | 35.39 | 95% effective doses | High | No |
| Lurasidone | 9.69 | 95% effective doses | High | No |
| Brexpiprazole | 0.22 | 95% effective doses | Partial agonist | Yes |
| Cariprazine | 0.50 | 95% effective doses | Partial agonist | Yes |
| Levomepromazine | 30 | DDD | High | Yes |

*Note:* Eq: equivalent dose; D2R: dopamine receptor 2; DDD: defined daily dose

Table S2. Characteristics of concomitant use of medications and blood cardiometabolic indices.

|  | Participants (n = 293) |
| --- | --- |
| *Characteristics of concomitant medication use*  AP polypharmacy, n (%)  Anti-depressants, n (%)  Benzodiazepines, n (%)  Mood stabilizer, n (%)  Anticholinergic medications, n (%)  hypoglycemic agents, n (%)  antihypertensive drugs, n (%)  *Cardiometabolic indices in blood*  Glucose, mean (SD)^a^  Triglycerides, mean (SD)^b^  Total Cholesterol, mean (SD)^b^  Low-density Cholesterol, mean (SD)^b^  High-density Cholesterol, mean (SD)^b^  Non-high-density Cholesterol, mean (SD)^b^ | 21 (7.2%)  33 (11.2%)  28 (9.6%)  9 (3.1%)  4 (1.4%)  3 (1.0%)  4 (1.4%)  5.3 (1.0)  1.3 (0.9)  4.9 (1.0)  3.0 (0.9)  1.3 (0.3)  3.6 (1.0) |

1. Available for 162 participants;
2. Available for 168 participants;

# **Comparisons between missing and non-missing data**

Table S3. Comparisons of participant characteristics between included and excluded patients.

| Variable | Included N = 293 (90.2%) | Excluded N = 32 (9.85%) | p-value |
| --- | --- | --- | --- |
| Sex(male), n (%) | 202 (68.9%) | 23 (71.9%) | 0.7^1^ |
| Age, mean (SD) | 28.40 (8.97) | 25.47 (7.44) | 0.058^2^ |
| Smoking (yes), n (%) | 110 (39.4%) | 16 (59.3%) | 0.046^1^ |
| Olanzapine equivalent dose, mean (SD) | 9.09 (5.21) | 8.33 (4.89) | 0.4^2^ |
| Metabolic risk (high), n (%)  APs D2R affinity  Low to medium  High  Partial agonist | 169 (57.7%)  132 (45.1%)  74 (25.3%)  87 (29.7%) | 16 (50.0%)  14 (43.8%)  12 (37.5%)  6 (18.8%) | 0.4^1^  0.2^1^ |
| BMI, mean (SD) | 25.13 (4.36) | 25.13 (4.52) | >0.9^2^ |
| Unknown | 15 | 3 |  |
| Waist circumference, mean (SD) | 92.34 (12.03) | 93.60 (13.71) | 0.9^2^ |
| Unknown | 56 | 11 |  |
| Parkinsonism SHRS mean scores, mean (SD) | 0.47 (0.53) | 0.38 (0.46) | 0.2^2^ |
| Unknown | 39 | 3 |  |
| Dyskinesia SHRS mean scores, mean (SD) | 0.10 (0.24) | 0.12 (0.27) | 0.6^2^ |
| Unknown | 30 | 3 |  |
| Akathisia SHRS mean scores, mean (SD) | 0.43 (0.62) | 0.38 (0.71) | 0.4^2^ |
| Unknown | 34 | 3 |  |
| Dystonia SHRS global score |  |  | 0.14^3^ |
| 0 | 226 (84.3%) | 21 (72.4%) |  |
| 1 | 11 (4.1%) | 3 (10.3%) |  |
| 2 | 18 (6.7%) | 2 (6.9%) |  |
| 3 | 11 (4.1%) | 2 (6.9%) |  |
| 4 | 2 (0.7%) | 1 (3.4%) |  |
| Unknown | 25 | 3 |  |
| Note: SD: standard deviation; APs: Antipsychotics; D2R: dopamine D2 receptor; SHRS: St. Hans Rating Scale;  ^1^Pearson's Chi-squared test; ^2^Wilcoxon rank sum test; ^3^Fisher's exact test | | | |

Table S4. Comparisons of characteristics between missing and non-missing participants in movement disorders.

|  | **Parkinsonism** | | | **Dyskinesia** | | | **Akathisia** | | | **Dystonia** | | |
| --- | --- | --- | --- | --- | --- | --- | --- | --- | --- | --- | --- | --- |
| **Variable** | **Not missing** N = 254 (86.7%)^1^ | **Missing** N = 39 (13.3%)^1^ | **p-value**^3^ | **Not missing** N = 263 (89.8%)^1^ | **Missing** N = 30 (10.2%)^1^ | **p-value**^3^ | **Not missing** N = 259 (88.4%)^1^ | **Missing** N = 34 (11.6%)^1^ | **p-value**^3^ | **Not missing** N = 268 (91.5%)^1^ | **Missing** N = 25 (8.53%)^1^ | **p-value**^3^ |
| **Sex (male), n (%)** | 176 (69.3%) | 26 (66.7%) | 0.7 | 182 (69.2%) | 20 (66.7%) | 0.8 | 181 (69.9%) | 21 (61.8%) | 0.3 | 185 (69.0%) | 17 (68.0%) | >0.9 |
| **Age, mean (SD)** | 28.48 (9.26) | 27.90 (6.88) | 0.7 | 28.43 (9.14) | 28.17 (7.37) | 0.7 | 28.35 (9.04) | 28.82 (8.52) | 0.5 | 28.40 (9.09) | 28.48 (7.70) | 0.6 |
| **Smoking (yes), n (%)** | 99 (41.1%) | 11 (28.9%) | 0.2 | 102 (40.8%) | 8 (27.6%) | 0.2 | 100 (40.7%) | 10 (30.3%) | 0.3 | 102 (40.2%) | 8 (32.0%) | 0.4 |
| **Olanzapine equivalent dose, mean (SD)** | 9.26 (5.30) | 7.96 (4.50) | 0.2 | 9.22 (5.23) | 7.97 (4.97) | 0.2 | 9.23 (5.27) | 8.00 (4.72) | 0.2 | 9.16 (5.21) | 8.37 (5.29) | 0.4 |
| **APs D2 affinity** |  |  | 0.3 |  |  | 0.2 |  |  | 0.11 |  |  | 0.12 |
| Low-medium | 111 (43.7%) | 21 (53.8%) |  | 114 (43.3%) | 18 (60.0%) |  | 111 (42.9%) | 21 (61.8%) |  | 116 (43.3%) | 16 (64.0%) |  |
| High | 68 (26.8%) | 6 (15.4%) |  | 68 (25.9%) | 6 (20.0%) |  | 68 (26.3%) | 6 (17.6%) |  | 69 (25.7%) | 5 (20.0%) |  |
| Partial antagonist | 75 (29.5%) | 12 (30.8%) |  | 81 (30.8%) | 6 (20.0%) |  | 80 (30.9%) | 7 (20.6%) |  | 83 (31.0%) | 4 (16.0%) |  |

Note: SD: standard deviation; APs: Antipsychotics; D2R: dopamine D2 receptor; SHRS: St. Hans Rating Scale;

^1^ Pearson’s Chi-squared test; ^2^ Wilcoxon rank sum test; ^3^ Fisher’s exact test

Table S5. Comparisons of characteristics between missing and non-missing participants in weight.

|  | **BMI** | | | **Waist Circumference** | | |
| --- | --- | --- | --- | --- | --- | --- |
| **Variable** | **Not missing** N = 278 (94.9%)^1^ | **Missing** N = 15 (5.12%)^1^ | **p-value**^3^ | **Not missing** N = 237 (80.9%)^1^ | **Missing** N = 56 (19.1%)^1^ | **p-value**^4^ |
| **Sex (male), n, (%)** | 191 (68.7%) | 11 (73.3%) | >0.9 | 163 (68.8%) | 39 (69.6%) | 0.9 |
| **Age, mean (SD)** | 28.38 (8.94) | 28.87 (9.78) | 0.9 | 28.16 (9.07) | 29.41 (8.53) | 0.13 |
| **Smoking (yes), n (%)** | 106 (40.0%) | 4 (28.6%) | 0.4 |  |  | 0.4 |
| **Olanzapine equivalent dose, mean (SD)** | 9.22 (5.24) | 6.59 (4.03) | 0.050* | 9.31 (5.11) | 8.16 (5.56) | 0.061 |
| **Metabolically active (high), n (%)** | 160 (57.6%) | 9 (60.0%) | 0.9 | 131 (55.3%) | 38 (67.9%) | 0.087 |

^1^ Fisher’s exact test; ^2^ Wilcoxon rank sum test; ^3^ Pearson’s Chi-squared test

# **Effect of antipsychotic-dose reduction stratified by D2R affinity and metabolic risk**

For movement disorders, we found that compared to low-medium D2R affinity AP, dose reduction of high D2R affinity AP was potentially more effective in decreasing mean parkinsonism scores (β = -.014, 95% CI -.030, .003, p = .092), but potentially associated with an increase in mean dyskinesia scores on the SHRS (β = .007, 95% CI -.000, .015, p = .070). Specifically, a 1 mg reduction in low-medium D2R AP olanzapine equivalent dose was associated with a .009-point decrease in mean parkinsonism scores (95% CI -.020, -.002, p = .059) and a .005-point decrease in mean tardive dyskinesia scores on the SHRS (95% CI -.009, -.001, p = .017). In contrast, a 1 mg in high D2R affinity AP olanzapine equivalent dose reduction was associated with a .023-point decrease in mean parkinsonism scores, but a .002-point increase in mean tardive dyskinesia scores. No interactions between AP reduced dose and D2R affinity group were found for akathisia and dystonia.

For cardiometabolic dysfunction, reducing the dose of more metabolically active AP was less effective in decreasing BMI (β = .056, 95% CI .012, .099, p = .012), compared to reducing less metabolically active AP dose. Specifically, a 1 mg reduction in less metabolically active AP olanzapine equivalent dose was associated with a .066 kg/m² decrease in BMI (95% CI: –0.099, –0.034, p < .001), whereas the decrease was only 0.01 kg/m² for more metabolically active AP. No interactions between AP reduced dose and metabolically active group were found for other cardiometabolic indices including systolic blood pressure, diastolic blood pressure, glucose, triglycerides, total cholesterol, HDL cholesterol, nonHDL cholesterol and LDL cholesterol. Please see Table S6-7 for detailed results.

Table S6. Effect of antipsychotic reduced dose magnitude and D2 affinity interaction on the change of movement disorders.

| Outcomes ^#^ | Variables | Estimates | 95% CI | P-value |
| --- | --- | --- | --- | --- |
| Parkinsonism ^a^ | Reduced dose | -.009 | -.020, -.002 | .059 |
|  | High D2-affinity | -.065 | -.194, .064 | .339 |
|  | D2 partial agonist | .029 | -.112, .167 | .694 |
|  | Reduced dose: High D2-affinity | -.014 | -.030, .003 | .092 |
|  | Reduced dose: D2 partial agonists | -.005 | -.018, .009 | .515 |
|  | Follow-up duration | .001 | -.002, .003 | .586 |
|  | *N* patients | 207 |  |  |
|  | *N* observations | 473 |  |  |
| Tardive dyskinesia ^a^ | Reduced dose | -.005 | -.009, -.001 | .017* |
|  | High D2-affinity | -.069 | -.117, -.022 | .006** |
|  | D2 partial agonist | -.004 | -.058, .048 | .873 |
|  | Reduced dose: High D2-affinity | .007 | -.000, .015 | .070 |
|  | Reduced dose: D2 partial agonists | .003 | -.003, .009 | .403 |
|  | Follow-up duration | -.001 | -.002, .001 | .332 |
|  | *N* patients | 215 |  |  |
|  | *N* observations | 493 |  |  |
| Akathisia ^a^ | Reduced dose | -.007 | -.018, .003 | .185 |
|  | High D2-affinity | -.026 | -.166, .115 | .720 |
|  | D2 partial agonist | -.004 | -.156, .147 | .957 |
|  | Reduced dose: High D2-affinity | .008 | -.012, .027 | .446 |
|  | Reduced dose: D2 partial agonists | .004 | -.011, .020 | .590 |
|  | Follow-up duration | .001 | -.002, .004 | .592 |
|  | *N* patients | 210 |  |  |
|  | *N* observations | 483 |  |  |
| Tardive dystonia ^a^ | Reduced Dose | -.007 | -.018, .003 | .185 |
|  | High D2-affinity | -.026 | -.166, .115 | .720 |
|  | D2 partial agonist | -.004 | -.156, .147 | .957 |
|  | Reduced dose: High D2-affinity | .008 | -.012, .027 | .446 |
|  | Reduced dose: D2 partial agonists | .004 | -.011, .020 | .590 |
|  | Follow-up duration | .001 | -.002, .004 | .592 |
|  | *N* patients | 210 |  |  |
|  | *N* observations | 483 |  |  |

1. Adjusted for follow-up duration, age, sex, smoking status, switch AP between different D2R affinity groups during follow-up, AP polypharmacy, concomitant use of other medications (antidepressants, benzodiazepines, mood stabilizers, anti-movement disorders medications) and baseline outcome values (baseline mean scores of parkinsonism, dyskinesia, akathisia and global dystonia score respectively)

Table S7. Effect of antipsychotic reduced dose magnitude and metabolically active interaction on the change of cardiometabolic indices.

| Outcomes^#^ | Variables | Estimates | 95% CI | P-value |
| --- | --- | --- | --- | --- |
| BMI ^a^ | Reduced dose | -.066 | -.099, -.034 | <.001 |
|  | Metabolically active | -.173 | -.642, .295 | .467 |
|  | Reduced dose: Metabolically active | .056 | .012, .099 | .012* |
|  | Follow-up duration | .019 | .012, .026 | <.001 |
|  | *N* patients | 260 |  |  |
|  | *N* observations | 863 |  |  |
| Waist circumference ^a^ | Reduced dose | -.138 | -.292, .016 | .078 |
|  | Metabolically active | -1.320 | -3.298, .658 | .190 |
|  | Reduced dose: Metabolically active | -.033 | -.257, .192 | .776 |
|  | Follow-up duration | .071 | .037, .105 | <.001*** |
|  | *N* patients | 200 |  |  |
|  | *N* observations | 494 |  |  |
| Systolic BP ^b^ | Reduced dose | .064 | -.177, .305 | .601 |
|  | Metabolically active | -.918 | -3.658, 1.821 | .510 |
|  | Reduced dose: Metabolically active | .157 | -.191, .505 | .376 |
|  | Follow-up duration | .071 | .015, .127 | .013* |
|  | *N* patients | 204 |  |  |
|  | *N* observations | 513 |  |  |
| Diastolic BP ^b^ | Reduced Dose | .131 | -.038, .300 | .128 |
|  | Metabolically active | .061 | -1.969, 2.092 | .952 |
|  | Reduced dose: Metabolically active | -.109 | -.354, .136 | .382 |
|  | Follow-up duration | .093 | .055, .132 | <.001*** |
|  | *N* patients | 204 |  |  |
|  | *N* observations | 513 |  |  |
| Glucose ^c^ | Reduced Dose | .044 | -.014, .103 | .137 |
|  | Metabolically active | .297 | -.316, .911 | .338 |
|  | Reduced dose: Metabolically active | -.046 | -.122, .029 | .229 |
|  | Follow-up duration | .044 | -.014, .103 | .137 |
|  | *N* patients | 86 |  |  |
|  | *N* observations | 103 |  |  |
| Triglycerides^d^ | Reduced Dose | -.020 | -.048, .007 | .149 |
|  | Metabolically active | -.162 | -.465, .142 | .294 |
|  | Reduced dose: Metabolically active | .021 | -.016, .058 | .262 |
|  | Follow-up duration | .011 | .003, .019 | .009** |
|  | *N* patients | 102 |  |  |
|  | *N* observations | 136 |  |  |
| Total Choles-terol ^d^ | Reduced Dose | -.018 | -.044, .007 | .153 |
|  | Metabolically active | -.161 | -.452, .130 | .274 |
|  | Reduced dose: Metabolically active | -.008 | -.042, .025 | .626 |
|  | Follow-up duration | .006 | -.001, .012 | .112 |
|  | *N* patients | 101 |  |  |
|  | *N* observations | 134 |  |  |
| HDL-Cholesterol ^d^ | Reduced Dose | -.008 | -.019, .004 | .179 |
|  | Metabolically active | -.104 | -.227, .019 | .096 |
|  | Reduced dose: Metabolically active | .009 | -.007, .024 | .262 |
|  | Follow-up duration | -.002 | -.005, .002 | .354 |
|  | *N* patients | 102 |  |  |
|  | *N* observations | 136 |  |  |
| LDL-Cholesterol ^d^ | Reduced Dose | -0.006 | -.029, .017 | .631 |
|  | Metabolically active | -.048 | -.312, .217 | .722 |
|  | Reduced dose: Metabolically active | -.022 | -.052, .009 | .166 |
|  | Follow-up duration | .001 | -.005, .007 | .766 |
|  | *N* patients | 102 |  |  |
|  | *N* observations | 136 |  |  |
| nonHDL- Cholesterol ^d^ | Reduced Dose | -.014 | -.041, .012 | .294 |
|  | Metabolically active | -.115 | -.423, .193 | .462 |
|  | Reduced dose: Metabolically active | -.012 | -.048, .024 | .503 |
|  | Follow-up duration | .005 | -.002, .012 | .195 |
|  | *N* patients | 102 |  |  |
|  | *N* observations | 136 |  |  |

*Note:* Abbreviations: AP: Antipsychotic; CI: Confidence interval; BMI: Body mass index; HDL: High-density lipoprotein; LDL: Low-density lipoprotein; BP: Blood pressure

^#^ For BMI, waist circumference, systolic BP, and diastolic BP, follow-up was conducted for up to 5 years. Glucose, TG, TC, HDL-C, LDL-C, and nonHDL-C were followed up for up to 4 years.

* P<0.05, ** P<0.01, *** P<0.001

a. Adjusted for follow-up duration, age, sex, smoking status, AP metabolic risk group, switch AP between different metabolic risk groups during follow-up, AP polypharmacy, concomitant use of other medications (antidepressants, benzodiazepines, mood stabilizers, lowering glucose, lowering fat, anti-hypertensive drugs), and baseline outcome values (baseline BMI, waist circumference respectively).

b. Adjusted for follow-up duration, age, sex, smoking status, AP metabolic risk group, switch AP between different metabolic risk groups during follow-up, AP polypharmacy, baseline outcome values, concomitant use of other medications (antidepressants, benzodiazepines, mood stabilizers, lowering fat, anti-hypertensive drugs), baseline systolic and diastolic blood pressure respectively.

c. Adjusted for follow-up duration, age, sex, smoking status, AP metabolic risk group, switch AP between different metabolic risk groups during follow-up, AP polypharmacy, baseline outcome values, fasting status, concomitant use of other medications, (antidepressants, benzodiazepines, mood stabilizers, anti-hypertensive drugs), and baseline glucose.

d. Adjusted for follow-up duration, age, sex, smoking status, AP metabolic risk group, switch AP between different metabolic risk groups during follow-up, AP polypharmacy, concomitant use of other medications, (antidepressants, benzodiazepines, mood stabilizers, anti-hypertensive drugs), baseline outcome values (baseline TG, TC, HDL-C, LDL-C, nonHDL-C respectively).

# **Effect of antipsychotic-dose reduction in patients with preexisting movement disorders and cardiometabolic dysfunction at baseline**

To explore if patients with movement disorders and cardiometabolic dysfunction at baseline can obtain a larger benefit from AP-dose reduction, we firstly identified *cases* of movement disorders and cardiometabolic dysfunction at baseline.

The movement disorders were identified by the following criteria:

***Parkinsonism*:** at least one of the following on SHRS parkinsonism subscale: (1) a rating of at least “mild” on the tremor or rigidity item; (2) “mild” on two items; or (3) “mild–moderate” on one item.^1^

***Dyskinesia*:** at least “mild–moderate” on one item or “mild” on two items on SHRS dyskinesia subscale.^1^

***Dystonia*:** at least “mild” on the global dystonia score.^2^

***Akathisia:*** at least “mild” on the Global Clinical Assessment of Akathisia.^3^

Cardiometabolic dysfunction was defined by the following thresholds:

***Overweight*:** BMI > 25 kg/m^2^;^4^

***Central obesity:*** Male ≥ 94 cm, Female ≥ 88 cm;^5^

***High systolic blood pressure****:* ≥ 130 mmHg;^5^

***High diastolic blood pressure:*** ≥ 85 mmHg;^5^

***Hyperglycemia****:* fasting glucose ≥ 5.6 mmol/L or non-fasting ≥ 7.8 mmol/L;^5^

***Hypertriglyceridemia****:*≥ 1.7 mmol/L;^5^

**Hypercholesteremia:** ≥ 5.2 mmol/L;^6^

***Low high-density lipoprotein cholesterol:*** male < 1.03 mmol/l, female < 1.29 mmol/l;^5^

***High low-density lipoprotein cholesterol:*** > 3.0 mmol/l;^7^

***High non-high-density lipoprotein cholesterol:*** > 3.8 mmol/l.^8^

We then conducted two additional analyses:

1. We introduced interaction terms between reduced AP olanzapine equivalent dose and the presence of each movement disorder and cardiometabolic dysfunction at baseline, respectively, to assess whether the effect of AP-dose reduction on changes in these adverse effects differed based on preexisting conditions;

2. We performed subgroup analyses including only patients with each movement disorder or cardiometabolic dysfunction at baseline, respectively, to examine whether the effect size of reduced AP olanzapine equivalent dose was larger in these subpopulations.

Regarding movement disorders, in our interaction models, we found that patients with preexisting parkinsonism experienced a greater effect of dose reduction on decreasing parkinsonism mean scores (β = -.016, 95% CI -.029, -.004, p = .011), as well as dyskinesia mean scores (β = -.014, 95% CI -.025, -.003, p = .013), compared to patients without parkinsonism and dyskinesia at baseline, respectively. In contrast, patients with preexisting dystonia showed a greater increase in dystonia scores following dose reduction (β = .031, 95% CI .005, .057, p = .022) than those without preexisting dystonia. Please see detailed results in supplementary Table S8.

In the subgroup of patients with movement disorders at baseline, we found that a 1 mg reduction in AP olanzapine equivalent dose was associated with a 0.017-point (95% -.026, -.007, p = .001) decrease in parkinsonism mean scores. No significant associations were found for tardive dyskinesia, akathisia, or tardive dystonia, which may be due to limited statistical power, as the baseline prevalence of tardive dyskinesia, tardive dystonia, and akathisia was low. Please see detailed results in supplementary Table S9.

These results indicate that patients with parkinsonism and dyskinesia at baseline may benefit more from dose reduction in mitigating them compared to those without these conditions at baseline. Although we found a significant interaction between reduced AP olanzapine equivalent dose and preexisting dystonia, AP-dose reduction was not associated with changes in dystonia scores in either the overall sample or among patients with preexisting dystonia. Therefore, it is difficult to conclude that AP-dose reduction has an effect on dystonia, regardless of its presence at baseline.

For cardiometabolic indices, In the interaction models, we found that, compared to patients without preexisting hyperglycemia, those with preexisting hyperglycemia showed a greater increase in glucose levels associated with AP-dose reduction (β = .121, 95% CI: .025 to 0.217, p =.014). In the subgroup analyses, a 1 mg reduction in olanzapine equivalent dose was associated with a 0.051 kg/m² decrease in BMI (95% CI: –.087 to –.016, p = .005), .026 mmol/L decrease in LDL-C (95% CI: –.051 to –.002, p =.038) and a potential 0.156 cm decrease in waist circumference (95% CI: –.325 to .013, p =.070). No significant associations were found for other cardiometabolic indices. The detailed results have been added to Supplementary Table S10-11.

Although we found a significant interaction between reduced AP olanzapine equivalent dose and baseline hyperglycemia, AP-dose reduction was not associated with changes in glucose levels in either the overall sample or in patients with baseline hyperglycemia. Therefore, it is difficult to conclude that AP-dose reduction has an effect on glucose levels, regardless of the presence of hyperglycemia at baseline. Additionally, while AP-dose reduction showed a larger effect size in reducing BMI and LDL-C among patients with baseline overweight and high LDL-C levels, these benefits were not significantly different from those observed in patients without these conditions.

Table S8. Effect of antipsychotic reduced dose and preexisting movement disorders at baseline interaction on the change of movement disorder measurements.

| Outcomes ^#^ | Variables | Estimates | 95% CI | P-value |
| --- | --- | --- | --- | --- |
| Parkinsonism ^a^ | Reduced dose | -.006 | -.015, .003 | .167 |
|  | Preexisting parkinsonism | -.461 | -.601, -.321 | <.001 |
|  | Reduced dose: Preexisting parkinsonism | -.016 | -.029, -.004 | .011* |
|  | Follow-up duration | .001 | -.001, .003 | .370 |
|  | *N* patients | 210 |  |  |
|  | *N* observations | 489 |  |  |
| Tardive dyskinesia ^a^ | Reduced dose | -.001 | -.005, .003 | .591 |
|  | Preexisting dyskinesia | -.546 | -.657, -.436 | <.001*** |
|  | Reduced dose: Preexisting dyskinesia | -.014 | -.025, -.003 | .013* |
|  | Follow-up duration | -.001 | -.002, .000 | .095 |
|  | *N* patients | 218 |  |  |
|  | *N* observations | 508 |  |  |
| Akathisia ^a^ | Reduced dose | -.004 | -.014, .005 | .336 |
|  | Preexisting akathisia | -1.047 | -1.238, -.855 | <.001 |
|  | Reduced dose: Preexisting akathisia | .005 | -.013, .023 | .595 |
|  | Follow-up duration | .001 | -.002, .004 | .603 |
|  | *N* patients | 213 |  |  |
|  | *N* observations | 499 |  |  |
| Tardive dystonia ^a^ | Reduced Dose | -.002 | -.012, .008 | .676 |
|  | Preexisting dystonia | -2.130 | -2.355, -1.906 | <.001 |
|  | Reduced dose: Preexisting dystonia | .031 | .005, .057 | .022* |
|  | Follow-up duration | .004 | .000, .008 | .053 |
|  | *N* patients | 226 |  |  |
|  | *N* observations | 540 |  |  |

*Note*: AP: Antipsychotic; CI: Confidence interval

* P<0.05, ** P<0.01, *** P<0.001

a. Adjusted for follow-up duration, age, sex, smoking status, AP D2R affinity groups, switch AP between different D2R affinity groups during follow-up, AP polypharmacy, concomitant use of other medications (antidepressants, benzodiazepines, mood stabilizers, anti-movement disorders medications) and baseline outcome values (baseline mean scores of parkinsonism, dyskinesia, akathisia and global dystonia score respectively)

Table S9. Effect of AP-dose reduction from baseline magnitude on changes in movement disorder measurements for patients with movement disorders at baseline subgroup over time.

| Outcomes ^#^ | Variables | Estimates | 95% CI | P-value |
| --- | --- | --- | --- | --- |
| Parkinsonism ^a^ | Reduced dose | -.017 | -.026, -.007 | .001** |
|  | Follow-up duration | -.002 | -.005, .002 | .350 |
|  | *N* patients | 93 |  |  |
|  | *N* observations | 216 |  |  |
| Tardive dyskinesia ^a^ | Reduced dose | -.002 | -.025, .021 | .878 |
|  | Follow-up duration | -.002 | -.011, .007 | .649 |
|  | *N* patients | 18 |  |  |
|  | *N* observations | 44 |  |  |
| Akathisia ^a^ | Reduced dose | .003 | -.025, .031 | .839 |
|  | Follow-up duration | .000 | -.011, .011 | .951 |
|  | *N* patients | 38 |  |  |
|  | *N* observations | 86 |  |  |
| Tardive dystonia ^a^ | Reduced Dose | .025 | -.011, .060 | .168 |
|  | Follow-up duration | .015 | -.006, .036 | .154 |
|  | *N* patients | 26 |  |  |
|  | *N* observations | 51 |  |  |

1. Adjusted for follow-up duration, age, sex, smoking status, switch AP between different D2R affinity groups during follow-up, AP polypharmacy, concomitant use of other medications (antidepressants, benzodiazepines, mood stabilizers, anti-movement disorders medications) and baseline outcome values (baseline mean scores of parkinsonism, dyskinesia, akathisia and global dystonia score respectively)

Table S10. Effect of antipsychotic reduced dose and preexisting cardiometabolic dysfunction at baseline interaction on the change of cardiometabolic indices.

| Outcomes^#^ | Variables | Estimates | 95% CI | P-value |
| --- | --- | --- | --- | --- |
| BMI ^a^ | Reduced dose | -.022 | -.052, .008 | .155 |
|  | Preexisting overweight | -.341 | -.824, .142 | .166 |
|  | Reduced dose: Preexisting overweight | -.014 | -.057, .028 | .506 |
|  | Follow-up duration | .017 | .010, .024 | <.001 |
|  | *N* patients | 263 |  |  |
|  | *N* observations | 881 |  |  |
| Waist circumference ^a^ | Reduced dose | -.198 | -.380, -.017 | .032* |
|  | Preexisting central obesity | -1.357 | -3.502, .787 | .213 |
|  | Reduced dose: Preexisting central obesity | .060 | -.172, .292 | .612 |
|  | Follow-up duration | .071 | .036, .105 | <.001 |
|  | *N* patients | 192 |  |  |
|  | *N* observations | 477 |  |  |
| Systolic BP ^b^ | Reduced dose | .219 | .004, .434 | .046* |
|  | Preexisting high systolic BP | -12.474 | -16.117, -8.832 | <.001 |
|  | Reduced dose: Preexisting systolic BP | -.270 | -.691, .152 | .209 |
|  | Follow-up duration | .078 | .020, .135 | .008** |
|  | *N* patients | 204 |  |  |
|  | *N* observations | 513 |  |  |
| Diastolic BP ^b^ | Reduced Dose | .100 | -.042, .241 | .166 |
|  | Preexisting high diastolic BP | -10.631 | -14.682, -6.580 | <.001 |
|  | Reduced dose: Preexisting diastolic BP | -.281 | -.687, .125 | .174 |
|  | Follow-up duration | .097 | .058, .135 | <.001 |
|  | *N* patients | 204 |  |  |
|  | *N* observations | 513 |  |  |
| Glucose ^c^ | Reduced Dose | -.004 | -.045, .036 | .830 |
|  | Preexisting hyperglycemia | -.690 | -1.498, .119 | .094 |
|  | Reduced dose: Preexisting hyperglycemia | .121 | .025, .217 | .014* |
|  | Follow-up duration | -.029 | -.059, .000 | .052 |
|  | *N* patients | 79 |  |  |
|  | *N* observations | 94 |  |  |
| Triglycerides ^d^ | Reduced Dose | -.016 | -.040, .008 | .203 |
|  | Preexisting hypertriglyceridemia | -.602 | -1.009, -0.195 | .004** |
|  | Reduced Dose: Preexisting hypertriglyceridemia | .019 | -.032, .070 | .463 |
|  | Follow-up duration | .015 | .006, .024 | .002** |
|  | *N* patients | 102 |  |  |
|  | *N* observations | 136 |  |  |
| Total Choles-terol ^d^ | Reduced Dose | -.018 | -.040, .004 | .100 |
|  | Preexisting hypercholesterolemia | -.341 | -.649, -.033 | .030* |
|  | Reduced Dose: Preexisting hypercholesterolemia | -.007 | -.042, .028 | .693 |
|  | Follow-up duration | .005 | -.002, .012 | .164 |
|  | *N* patients | 101 |  |  |
|  | *N* observations | 134 |  |  |
| HDL-Cholesterol ^d^ | Reduced Dose | -.001 | -.010, .008 | .808 |
|  | Preexisting hypoalphalipoproteinemia | .079 | -.062, .221 | .269 |
|  | Reduced Dose: Preexisting hypoalphalipoproteinemia | .001 | -.019, .021 | .937 |
|  | Follow-up duration | -.001 | -.004, .002 | .495 |
|  | *N* patients | 100 |  |  |
|  | *N* observations | 133 |  |  |
| LDL-Cholesterol ^d^ | Reduced Dose | -.006 | -.029, .017 | .583 |
|  | Preexisting high LDL-C | -.146 | -.422, .130 | .297 |
|  | Reduced Dose: Preexisting high LDL-C | -.017 | -.048, .013 | .271 |
|  | Follow-up duration | .000 | -.006, .006 | .994 |
|  | *N* patients | 102 |  |  |
|  | *N* observations | 136 |  |  |
| nonHDL- Cholesterol ^d^ | Reduced Dose | -.018 | -.039, .003 | .091 |
|  | Preexisting high nonHDL-C | -.440 | -.778, -.101 | .011 |
|  | Reduced Dose: Preexisting high nonHDL-C | -.004 | -.045, .037 | .832 |
|  | Follow-up duration | .004 | -.003, .011 | .251 |
|  | *N* patients | 102 |  |  |
|  | *N* observations | 136 |  |  |

*Note:* Abbreviations: AP: Antipsychotic; CI: Confidence interval; BMI: Body mass index; HDL: High-density lipoprotein; LDL: Low-density lipoprotein; BP: Blood pressure

^#^ For BMI, waist circumference, systolic BP, and diastolic BP, follow-up was conducted for up to 5 years. Glucose, TG, TC, HDL-C, LDL-C, and nonHDL-C were followed up for up to 4 years.

* P<0.05, ** P<0.01, *** P<0.001

a. Adjusted for follow-up duration, age, sex, smoking status, AP metabolic risk group, switch AP between different metabolic risk groups during follow-up, AP polypharmacy, concomitant use of other medications (antidepressants, benzodiazepines, mood stabilizers, lowering glucose, lowering fat, anti-hypertensive drugs), and preexisting overweight, central obesity respectively at baseline.

b. Adjusted for follow-up duration, age, sex, smoking status, AP metabolic risk group, switch AP between different metabolic risk groups during follow-up, AP polypharmacy, baseline outcome values, concomitant use of other medications (antidepressants, benzodiazepines, mood stabilizers, lowering fat, anti-hypertensive drugs), preexisting hyper-systolic BP, and hyper-diastolic BP respectively at baseline.

c. Adjusted for follow-up duration, age, sex, smoking status, AP metabolic risk group, switch AP between different metabolic risk groups during follow-up, AP polypharmacy, baseline outcome values, fasting status, concomitant use of other medications, (antidepressants, benzodiazepines, mood stabilizers, anti-hypertensive drugs), and preexisting hyperglycemia.

d. Adjusted for follow-up duration, age, sex, smoking status, AP metabolic risk group, switch AP between different metabolic risk groups during follow-up, AP polypharmacy, concomitant use of other medications, (antidepressants, benzodiazepines, mood stabilizers, anti-hypertensive drugs), preexisting hypertriglyceridemia, hypercholesterolemia, low HDL-C, high LDL-C, high nonHDL-C respectively.

Table S11. Effect of AP-dose reduction from baseline magnitude on changes in cardiometabolic indices for patients with preexisting cardiometabolic dysfunction at baseline subgroup over time.

| Outcomes^#^ | Variables | Estimates | 95% CI | P-value |
| --- | --- | --- | --- | --- |
| BMI ^a^ | Reduced dose | -.051 | -.087, -.016 | .005** |
|  | Follow-up duration | .023 | .010, .036 | .001** |
|  | *N* patients | 105 |  |  |
|  | *N* observations | 336 |  |  |
| Waist circumference ^a^ | Reduced dose | -.156 | -.325, .013 | .070 |
|  | Follow-up duration | .100 | .049, .152 | <.001*** |
|  | *N* patients | 101 |  |  |
|  | *N* observations | 254 |  |  |
| Systolic BP ^b^ | Reduced dose | -.017 | -.364, .331 | .924 |
|  | Follow-up duration | -.033 | -.153, .087 | .590 |
|  | *N* patients | 49 |  |  |
|  | *N* observations | 112 |  |  |
| Diastolic BP ^b^ | Reduced Dose | -.189 | -.614, .237 | .373 |
|  | Follow-up duration | .070 | -.054, .193 | .259 |
|  | *N* patients | 18 |  |  |
|  | *N* observations | 45 |  |  |
| Glucose ^c^ | Reduced Dose | .622 | -.449, 1.694 | .130 |
|  | Follow-up duration | -.154 | -.623, .315 | .293 |
|  | *N* patients | 10 |  |  |
|  | *N* observations | 14 |  |  |
| Triglycerides^d^ | Reduced Dose | .001 | -.088, .091 | .974 |
|  | Follow-up duration | .033 | -.004, .070 | .075 |
|  | *N* patients | 22 |  |  |
|  | *N* observations | 32 |  |  |
| Total Choles-terol ^d^ | Reduced Dose | -.029 | -.065, .008 | .117 |
|  | Follow-up duration | .001 | -.015, .017 | .873 |
|  | *N* patients | 33 |  |  |
|  | *N* observations | 44 |  |  |
| HDL-Cholesterol ^d^ | Reduced Dose | -.003 | -.015, .008 | .573 |
|  | Follow-up duration | -.002 | -.004, .001 | .240 |
|  | *N* patients | 32 |  |  |
|  | *N* observations | 46 |  |  |
| LDL-Cholesterol ^d^ | Reduced Dose | -.026 | -.051, -.002 | .038* |
|  | Follow-up duration | -.003 | -.013, .007 | .583 |
|  | *N* patients | 47 |  |  |
|  | *N* observations | 62 |  |  |
| nonHDL- Cholesterol ^d^ | Reduced Dose | -.023 | -.073, .027 | .355 |
|  | Follow-up duration | .002 | -.013, .017 | .781 |
|  | *N* patients | 34 |  |  |
|  | *N* observations | 44 |  |  |

*Note:* Abbreviations: AP: Antipsychotic; CI: Confidence interval; BMI: Body mass index; HDL: High-density lipoprotein; LDL: Low-density lipoprotein; BP: Blood pressure

^#^ For BMI, waist circumference, systolic BP, and diastolic BP, follow-up was conducted for up to 5 years. Glucose, TG, TC, HDL-C, LDL-C, and nonHDL-C were followed up for up to 4 years.

* P<0.05, ** P<0.01, *** P<0.001

a. Adjusted for follow-up duration, age, sex, smoking status, AP metabolic risk group, switch AP between different metabolic risk groups during follow-up, AP polypharmacy, concomitant use of other medications (antidepressants, benzodiazepines, mood stabilizers, lowering glucose, lowering fat, anti-hypertensive drugs), and baseline outcome values (baseline BMI, waist circumference respectively).

b. Adjusted for follow-up duration, age, sex, smoking status, AP metabolic risk group, switch AP between different metabolic risk groups during follow-up, AP polypharmacy, baseline outcome values, concomitant use of other medications (antidepressants, benzodiazepines), baseline systolic and diastolic blood pressure respectively.

c. Adjusted for follow-up duration, age, sex, smoking status, AP metabolic risk group, switch AP between different metabolic risk groups during follow-up, AP polypharmacy, baseline outcome values, fasting status, concomitant use of other medications, (antidepressants, anti-hypertensive drugs), and baseline glucose.

d. Adjusted for follow-up duration, age, sex, smoking status, AP metabolic risk group, switch AP between different metabolic risk groups during follow-up, AP polypharmacy, concomitant use of other medications (antidepressants, benzodiazepines, mood stabilizers, anti-hypertensive drugs), baseline outcome values (baseline TG, HDL-C respectively).

# **Detailed results on the effect of antipsychotic-dose reduction from baseline magnitude on defined movement disorder cases**

Table S12. Effect of AP-dose reduction from baseline magnitude on the odds of movement disorders presence over time.

| Outcomes ^#^ | Variables | Odds Ratio | 95% CI | P-value |
| --- | --- | --- | --- | --- |
| Parkinsonism ^a^ | Reduced dose | 0.911 | 0.872, 0.953 | <.001** |
|  | Follow-up duration | 1.014 | 0.997, 1.032 | .096 |
|  | *N* patients | 211 |  |  |
|  | *N* observations | 504 |  |  |
| Tardive dyskinesia ^a^ | Reduced dose | 0.940 | 0.878, 1.006 | .074 |
|  | Follow-up duration | 0.999 | 0.965, 1.034 | .949 |
|  | *N* patients | 215 |  |  |
|  | *N* observations | 493 |  |  |
| Akathisia ^a^ | Reduced dose | 0.955 | 0.894, 1.020 | .170 |
|  | Follow-up duration | 1.027 | 1.001, 1.055 | .044 |
|  | *N* patients | 212 |  |  |
|  | *N* observations | 496 |  |  |
| Tardive dystonia ^a^ | Reduced Dose | 0.995 | 0.920, 1.076 | .901 |
|  | Follow-up duration | 1.032 | 0.998, 1.066 | .064 |
|  | *N* patients | 216 |  |  |
|  | *N* observations | 511 |  |  |

1. Adjusted for follow-up duration, age, sex, smoking status, switch AP between different D2R affinity groups during follow-up, AP polypharmacy, concomitant use of other medications (antidepressants, benzodiazepines, mood stabilizers, anti-movement disorders medications) and baseline outcome values (baseline mean scores of parkinsonism, dyskinesia, akathisia and global dystonia score respectively)

# **Models adjusted for substance use**

We included the variables including average weekly alcohol intake over the past month (median = 1, IQR = 3), frequency of hard drug use in the past month (median = 0, IQR = 0) and frequency of soft drug use in the past month (median = 0, IQR = 0) as covariates in the mixed-effects models, and re-ran the analyses.

The results show that a 1 mg reduction in olanzapine-equivalent dose was associated with decreases in parkinsonism scores by 0.013 (95% CI = -0.019, -0.006, p < .001), tardive dyskinesia scores by 0.003 (95% CI = -0.006, -0.001, p = .025), BMI by 0.041 kg/m² (95% CI = -0.064, -0.018, p < .001), waist circumference by 0.144 cm (95% CI = -0.260, -0.026, p = .179), total cholesterol by 0.020 mmol/L (95% CI = -0.035, -0.005, p = .018), LDL-C by 0.017 mmol/L (95% CI = -0.031, -0.003, p = .029), and non-HDL-C by 0.018 mmol/L (95% CI = -0.034, -0.002, p = 0.040). We found no associations for akathisia, tardive dystonia, systolic or diastolic blood pressure, glucose, triglycerides, or HDL-C. See table S13-14 for detailed results.

Table S13. Effect of AP-dose reduction from baseline magnitude on changes in movement disorder measurements over time, after further adjusted substance use.

| Outcomes ^#^ | Variables | Estimates | 95% CI | P-value |
| --- | --- | --- | --- | --- |
| Parkinsonism ^a^ | Reduced dose | -.013 | -.019, -.006 | <.001*** |
|  | Follow-up duration | .001 | -.002, .003 | .623 |
|  | *N* patients | 203 |  |  |
|  | *N* observations | 453 |  |  |
| Tardive dyskinesia ^a^ | Reduced dose | -.003 | -.006, .000 | .025* |
|  | Follow-up duration | -.001 | -.002, .001 | .260 |
|  | *N* patients | 211 |  |  |
|  | *N* observations | 473 |  |  |
| Akathisia ^a^ | Reduced dose | -.004 | -.012, .003 | .269 |
|  | Follow-up duration | .001 | -.001, .004 | .347 |
|  | *N* patients | 206 |  |  |
|  | *N* observations | 462 |  |  |
| Tardive dystonia ^a^ | Reduced Dose | .000 | -.009, .009 | .942 |
|  | Follow-up duration | .003 | .000, .007 | .086 |
|  | *N* patients | 219 |  |  |
|  | *N* observations | 500 |  |  |

1. Adjusted for follow-up duration, age, sex, smoking status, switch AP between different D2R affinity groups during follow-up, AP polypharmacy, use of substance (average weekly alcohol intake in the past month, frequency of hard drug use, and frequency of soft drug use in the past month), concomitant use of other medications (antidepressants, benzodiazepines, mood stabilizers, anti-movement disorders medications) and baseline outcome values (baseline mean scores of parkinsonism, dyskinesia, akathisia and global dystonia score respectively)

Table S14. Effect of AP-dose reduction from baseline magnitude on changes in cardiometabolic indices over time (adjusted substance use).

| Outcomes^#^ | Variables | Estimates | 95% CI | P-value |
| --- | --- | --- | --- | --- |
| BMI ^a^ | Reduced dose | -.041 | -.065, -.018 | .001** |
|  | Follow-up duration | .019 | .012, .026 | <.001*** |
|  | *N* patients | 258 |  |  |
|  | *N* observations | 827 |  |  |
| Waist circumference ^a^ | Reduced dose | -.144 | -.263, -.025 | .018* |
|  | Follow-up duration | .075 | .040, .109 | <.001*** |
|  | *N* patients | 195 |  |  |
|  | *N* observations | 474 |  |  |
| Systolic BP ^b^ | Reduced dose | .080 | -.103, .264 | .390 |
|  | Follow-up duration | .063 | .006, .120 | .031* |
|  | *N* patients | 200 |  |  |
|  | *N* observations | 494 |  |  |
| Diastolic BP ^b^ | Reduced Dose | .056 | -.074, .185 | .396 |
|  | Follow-up duration | .087 | .049, .126 | <.001 |
|  | *N* patients | 200 |  |  |
|  | *N* observations | 494 |  |  |
| Glucose ^c^ | Reduced Dose | .021 | -.015, .058 | .246 |
|  | Follow-up duration | -.025 | -.053, .004 | .093 |
|  | *N* patients | 83 |  |  |
|  | *N* observations | 100 |  |  |
| Triglycerides^d^ | Reduced Dose | -.006 | -.024, .013 | .540 |
|  | Follow-up duration | .010 | .002, .018 | .017* |
|  | *N* patients | 97 |  |  |
|  | *N* observations | 129 |  |  |
| Total Choles-terol ^d^ | Reduced Dose | -.020 | -.036, -.003 | .018* |
|  | Follow-up duration | .002 | -.005, .009 | .558 |
|  | *N* patients | 96 |  |  |
|  | *N* observations | 127 |  |  |
| HDL-Cholesterol ^d^ | Reduced Dose | -.004 | -.012, .004 | .362 |
|  | Follow-up duration | -.001 | -.004, .003 | .651 |
|  | *N* patients | 97 |  |  |
|  | *N* observations | 129 |  |  |
| LDL-Cholesterol ^d^ | Reduced Dose | -.017 | -.033, -.002 | .029* |
|  | Follow-up duration | -.002 | -.008, .005 | .579 |
|  | *N* patients | 97 |  |  |
|  | *N* observations | 129 |  |  |
| nonHDL- Cholesterol ^d^ | Reduced Dose | -.018 | -.036, -.001 | .040* |
|  | Follow-up duration | .000 | -.007, .008 | .902 |
|  | *N* patients | 97 |  |  |
|  | *N* observations | 129 |  |  |

*Note:* Abbreviations: AP: Antipsychotic; CI: Confidence interval; BMI: Body mass index; HDL: High-density lipoprotein; LDL: Low-density lipoprotein; BP: Blood pressure

^#^ For BMI, waist circumference, systolic BP, and diastolic BP, follow-up was conducted for up to 5 years. Glucose, TG, TC, HDL-C, LDL-C, and nonHDL-C were followed up for up to 4 years.

* P<0.05, ** P<0.01, *** P<0.001

a. Adjusted for follow-up duration, age, sex, smoking status, AP metabolic risk group, switch AP between different metabolic risk groups during follow-up, AP polypharmacy, use of substance (average weekly alcohol intake in the past month, frequency of hard drug use, and frequency of soft drug use in the past month), concomitant use of other medications (antidepressants, benzodiazepines, mood stabilizers, lowering glucose, lowering fat, anti-hypertensive drugs), and baseline outcome values (baseline BMI, waist circumference respectively).

b. Adjusted for follow-up duration, age, sex, smoking status, AP metabolic risk group, switch AP between different metabolic risk groups during follow-up, AP polypharmacy, use of substance (average weekly alcohol intake in the past month, frequency of hard drug use, and frequency of soft drug use in the past month), baseline outcome values, concomitant use of other medications (antidepressants, benzodiazepines, mood stabilizers, lowering fat, anti-hypertensive drugs), baseline systolic and diastolic blood pressure respectively.

c. Adjusted for follow-up duration, age, sex, smoking status, AP metabolic risk group, switch AP between different metabolic risk groups during follow-up, AP polypharmacy, use of substance (average weekly alcohol intake in the past month, frequency of hard drug use, and frequency of soft drug use in the past month), baseline outcome values, fasting status, concomitant use of other medications, (antidepressants, benzodiazepines, mood stabilizers, anti-hypertensive drugs), and baseline glucose.

d. Adjusted for follow-up duration, age, sex, smoking status, AP metabolic risk group, switch AP between different metabolic risk groups during follow-up, AP polypharmacy, use of substance (average weekly alcohol intake in the past month, frequency of hard drug use, and frequency of soft drug use in the past month), concomitant use of other medications, (antidepressants, benzodiazepines, mood stabilizers, anti-hypertensive drugs), baseline outcome values (baseline TG, TC, HDL-C, LDL-C, nonHDL-C respectively).

# **Time-lagged antipsychotic-dose reduction analyses**

**Method**

AP-dose reduction was calculated using time-lagged dose reduction: calculated by subtracting the dose at each visit from the dose at previous visit. To facilitate interpretation, dose reduction values were recentered by multiplying by -1. Movement disorder assessments and cardiometabolic indices were also calculated as time-lagged differences.

The relationship between time-lagged AP-dose reduction and time-lagged changes in movement disorders and cardiometabolic indices was evaluated using mixed-effect models. *time-lagged AP-dose reduction* as the independent variable as well as time-lagged movement disorders and cardiometabolic indices changes as the dependent variables. Baseline observations were removed when modeling, as no dose reduction occurred at baseline.

**Results**

Time-lagged AP-dose reduction was related to a time-lagged decrease in parkinsonism and tardive dyskinesia (β = -.011, 95% CI -.020, -.002 and β = -.006, 95% CI -.012, -.001, respectively), but we found no evidence for a relation with akathisia or tardive dystonia, meaning that time-lagged dose reduction was associated with decreases in parkinsonism and dyskinesia. More specifically, 1 mg of time-lagged AP-dose reduction was associated with decreases in parkinsonism and tardive dyskinesia by .011 and .006 respectively (Table S15).

No associations were found between time-lagged AP-dose reduction and time-lagged changes in any cardiometabolic indices, including BMI, waist circumference, systolic BP, diastolic BP, glucose, TG, TC, HDL-C, LDL-C, or nonHDL-C (Table S16).

Table S15. Effect of time-lagged AP-dose reduction magnitude on changes in movement disorder measurements over time

| Outcomes ^#^ | Variables | Estimates | 95% CI | P-value |
| --- | --- | --- | --- | --- |
| Parkinsonism ^a^ | Reduced dose | -.011 | -.020, -.002 | .021* |
|  | Follow-up duration | .003 | -.000, .007 | .075 |
|  | *N* patients | 186 |  |  |
|  | *N* observations | 376 |  |  |
| Tardive dyskinesia ^a^ | Reduced dose | -.006 | -.012, -.001 | .028* |
|  | Follow-up duration | .000 | -.002, .002 | .790 |
|  | *N* patients | 193 |  |  |
|  | *N* observations | 395 |  |  |
| Akathisia ^a^ | Reduced dose | .002 | -.010, .013 | .746 |
|  | Follow-up duration | .000 | -.004, .005 | .844 |
|  | *N* patients | 191 |  |  |
|  | *N* observations | 383 |  |  |
| Tardive dystonia ^a^ | Reduced Dose | .002 | -.012, .016 | .764 |
|  | Follow-up duration | .008 | .002, .013 | .007** |
|  | *N* patients | 202 |  |  |
|  | *N* observations | 435 |  |  |

*Note*: AP: Antipsychotic; CI: Confidence interval

^#^ Mixed effects models using time-lagged AP-dose reduction (calculated by subtracting the olanzapine equivalent dose at each visit from the dose at previous visit) as the independent variable and time-lagged movement disorders changes as the dependent variables.

* P<0.05, ** P<0.01, *** P<0.001

a. Adjusted for follow-up duration, age, sex, smoking status, AP D2R affinity groups, switch AP between different D2R affinity groups during follow-up, AP polypharmacy, concomitant use of other medications (antidepressants, benzodiazepines, mood stabilizers, anti-movement disorders medications) and baseline outcome values (baseline mean scores of parkinsonism, dyskinesia, akathisia and global dystonia score respectively)

Table S16. Effect of time-lagged AP-dose reduction magnitude on changes in cardiometabolic indices over time

| Outcomes^#^ | Variables | Estimates | 95% CI | P-value |
| --- | --- | --- | --- | --- |
| BMI ^a^ | Reduced dose | -.014 | -.036, .009 | .247 |
|  | Follow-up duration | .005 | -.003, .012 | .203 |
|  | *N* patients | 250 |  |  |
|  | *N* observations | 759 |  |  |
| Waist circumference ^a^ | Reduced dose | -.034 | -.178, .110 | .651 |
|  | Follow-up duration | .019 | -.032, .069 | .476 |
|  | *N* patients | 156 |  |  |
|  | *N* observations | 353 |  |  |
| Systolic BP ^b^ | Reduced dose | .088 | -.199, 375 | .556 |
|  | Follow-up duration | -.023 | -.117, .071 | .633 |
|  | *N* patients | 158 |  |  |
|  | *N* observations | 366 |  |  |
| Diastolic BP ^b^ | Reduced Dose | .070 | -.136, .277 | .512 |
|  | Follow-up duration | .024 | -.043, .091 | .495 |
|  | *N* patients | 158 |  |  |
|  | *N* observations | 366 |  |  |
| Glucose ^c^ | Reduced Dose | .078 | -.013, .168 | .128 |
|  | Follow-up duration | -.041 | -.084, .002 | .090 |
|  | *N* patients | 71 |  |  |
|  | *N* observations | 85 |  |  |
| Triglycerides ^d^ | Reduced Dose | -.015 | -.058, .027 | .516 |
|  | Follow-up duration | .009 | -.002, .021 | .132 |
|  | *N* patients | 81 |  |  |
|  | *N* observations | 103 |  |  |
| Total Choles-terol ^d^ | Reduced Dose | -.016 | -.051, .018 | .390 |
|  | Follow-up duration | .013 | .004, .022 | .011* |
|  | *N* patients | 80 |  |  |
|  | *N* observations | 102 |  |  |
| HDL-Cholesterol ^d^ | Reduced Dose | -.001 | -.016, .014 | .897 |
|  | Follow-up duration | .005 | .001, .009 | .035* |
|  | *N* patients | 81 |  |  |
|  | *N* observations | 103 |  |  |
| LDL-Cholesterol ^d^ | Reduced Dose | -.010 | -.042, .021 | .538 |
|  | Follow-up duration | .003 | -.005, .011 | .476 |
|  | *N* patients | 81 |  |  |
|  | *N* observations | 103 |  |  |
| nonHDL- Cholesterol ^d^ | Reduced Dose | -.017 | -.053, .018 | .371 |
|  | Follow-up duration | .006 | -.003, .015 | .253 |
|  | *N* patients | 81 |  |  |
|  | *N* observations | 103 |  |  |

*Note:* Abbreviations: AP: Antipsychotic; CI: Confidence interval; BMI: Body mass index; HDL: High-density lipoprotein; LDL: Low-density lipoprotein; BP: Blood pressure

^#^ Mixed effects models using time-lagged AP-dose reduction (calculated by subtracting the olanzapine equivalent dose at each visit from the dose at previous visit) as the independent variable and time-lagged cardiometabolic indices changes as the dependent variables; For BMI, waist circumference, systolic BP, and diastolic BP, follow-up was conducted for up to 5 years. Glucose, TG, TC, HDL-C, LDL-C, and nonHDL-C were followed up for up to 4 years.

* P<0.05, ** P<0.01, *** P<0.001

a. Adjusted for follow-up duration, age, sex, smoking status, AP metabolic risk group, switch AP between different metabolic risk groups during follow-up, AP polypharmacy, concomitant use of other medications (antidepressants, benzodiazepines, mood stabilizers, lowering glucose, lowering fat, anti-hypertensive drugs), and baseline outcome values (baseline BMI, waist circumference respectively).

b. Adjusted for follow-up duration, age, sex, smoking status, AP metabolic risk group, switch AP between different metabolic risk groups during follow-up, AP polypharmacy, baseline outcome values, concomitant use of other medications (antidepressants, benzodiazepines, mood stabilizers, lowering fat, anti-hypertensive drugs), baseline systolic and diastolic blood pressure respectively.

c. Adjusted for follow-up duration, age, sex, smoking status, AP metabolic risk group, switch AP between different metabolic risk groups during follow-up, AP polypharmacy, baseline outcome values, fasting status, concomitant use of other medications, (antidepressants, benzodiazepines, mood stabilizers, anti-hypertensive drugs), and baseline glucose.

d. Adjusted for follow-up duration, age, sex, smoking status, AP metabolic risk group, switch AP between different metabolic risk groups during follow-up, AP polypharmacy, concomitant use of other medications, (antidepressants, benzodiazepines, mood stabilizers, anti-hypertensive drugs), baseline outcome values (baseline TG, TC, HDL-C, LDL-C, nonHDL-C respectively).

# **The association between antipsychotic dose and movement disorder and cardiometabolic indices**

**Method**

We evaluated the relationships between AP dose and mean scores of each movement disorder scale and cardiometabolic indices. Due to the excess of zero scores on the movement disorders scales, we used two-part mixed-effects models: a generalized mixed-effects model for estimating the probability of zero scores and a linear mixed-effects model for non-zero scores.^9^ At each visit, AP dose was the independent variable, and the mean score of each movement disorder scale and cardiometabolic indices were the dependent variables.

**Results**

AP dose was negatively related to the odds of zero scores for parkinsonism and tardive dyskinesia and positively with the part of non-zero scores. Specifically, we found that 1mg olanzapine equivalent dose increase was associated with a decrease of 12% odds (OR = 0.881, 95% CI 0.845, 0.918, p < .001) in absent of parkinsonism, 4% odds (OR = 0.960, 95% CI 0.925, 0.997, p = .035) in absent of dyskinesia. Additionally, 1 mg olanzapine equivalent dose increase was associated with an increase of 0.008 points (β = 0.008, 95% CI 0.002, 0.014, p = .014) of parkinsonism severity, and 0.009 points (β = 0.009, 95% CI 0.001, 0.016, p = .044) of dyskinesia severity on the SHRS. We found no evidence for an association with akathisia or tardive dystonia (Table S17).

We found that AP dose was positively associated with BMI, waist circumference, TG, TC, LDL-C, and nonHDL-C. Specifically, 1 mg antipsychotic olanzapine equivalent dose increase was associated with increases of 0.036 kg/m^2^ (β = 0.036, 95% CI 0.018, 0.054, p < .001) in BMI, 0.142 cm (β = 0.142, 95% CI 0.063, 0.222, p < .001) in waist circumference, 0.019 mmol/L (β = 0.019, 95% CI 0.002, 0.023, p = .023) in triglycerides, 0.017 mmol/L (β = 0.017, 95% CI 0.008, 0.025, p < .001) in TC, 0.013 mmol/L (β = 0.013, 95% CI 0.005, 0.021, p = .001) in LDL-C, and 0.018 mmol/L (β = 0.018, 95% CI 0.009, 0.026, p < .001) in nonHDL-C. We found no evidence that AP dose was associated with BP, glucose or HDL-C (Table S18).

Table S17. Effects of AP dose on movement disorders over time.

| Outcomes^#^ |  | Variables | Estimates^##^ | 95% CI | P-value |
| --- | --- | --- | --- | --- | --- |
| Parkinsonism ^a^ | *Part 1* | Dose | 0.881 | 0.845, 0.918 | <.001*** |
|  |  | Follow-up duration | 1.005 | 0.991, 1.019 | .469 |
|  | *Part 2* | Dose | 0.008 | 0.002, 0.014 | .014* |
|  |  | Follow-up duration | 0.001 | -0.002, 0.003 | .692 |
|  |  | *N* patients | 245 |  |  |
|  |  | *N* observations | 711 |  |  |
| Dyskinesia ^a^ | *Part 1* | Dose | 0.960 | 0.925, 0.997 | .035* |
|  |  | Follow-up duration | 1.019 | 1.001, 1.037 | .040* |
|  | *Part 2* | Dose | 0.009 | 0.001, 0.016 | .044* |
|  |  | Follow-up duration | 0.001 | -0.003, 0.005 | .624 |
|  |  | *N* patients | 254 |  |  |
|  |  | *N* observations | 740 |  |  |
| Akathisia ^a^ | *Part 1* | Dose | 0.987 | 0.956, 1.018 | .409 |
|  |  | Follow-up duration | 0.996 | 0.983, 1.009 | .514 |
|  | *Part 2* | Dose | 0.006 | -0.003, 0.015 | .169 |
|  |  | Follow-up duration | 0.002 | -0.002, 0.006 | .354 |
|  |  | *N* patients | 250 |  |  |
|  |  | *N* observations | 726 |  |  |
| Dystonia ^a^ | *Part 1* | Dose | 1.004 | 0.968, 1.042 | .826 |
|  |  | Follow-up duration | 0.988 | 0.972, 1.005 | .161 |
|  | *Part 2* | Dose | -0.000 | -0.024, 0.021 | .968 |
|  |  | Follow-up duration | 0.009 | -0.001, 0.018 | .104 |
|  |  | *N* patients | 259 |  |  |
|  |  | *N* observations | 776 |  |  |

*Note*: AP: Antipsychotic; CI: Confidence interval

# Two-part mixed effects models: Part 1: Generalized linear mixed binary models using olanzapine equivalent dose as the independent variable, and zero or non-zero mean scores of movement disorder scales (binary, 1 = zero mean scores, 0 = non-zero scores) as the dependent variables, Part 2: Linear mixed models, using olanzapine equivalent dose as the independent variable, and non-zero mean scores of movement disorder scales (continuous) as the dependent variables

^##^ In the part 1 models, the estimates are odds ratios; In the part 2 models, the estimates are β coefficients.

* P<0.05, ** P<0.01, *** P<0.001

^a.^ Adjusted for follow-up duration, age, sex, smoking status, APs D2R affinity groups, switch APs between different D2R affinity groups during follow-up, APs polypharmacy, concomitant use of other medications (antidepressants, benzodiazepines, mood stabilizers, anti-movement disorders medications), baseline mean parkinsonism, dyskinesia, akathisia and global dystonia respectively

Table S18. Effects of AP dose on cardiometabolic indices over time.

| Outcomes^#^ | Variables | Estimates | 95% CI | P-value |
| --- | --- | --- | --- | --- |
| BMI ^a^ | Dose | .036 | .018, .054 | <.001*** |
|  | Follow-up duration | .023 | .017, .029 | <.001*** |
|  | *N* patients | 272 |  |  |
|  | *N* observations | 1125 |  |  |
| Waist circumference ^a^ | Dose | .142 | .063, .222 | <.001*** |
|  | Follow-up duration | .080 | .052, .108 | <.001*** |
|  | *N* patients | 230 |  |  |
|  | *N* observations | 717 |  |  |
| Systolic BP ^a^ | Dose | -.113 | -.242, .015 | .091 |
|  | Follow-up duration | .060 | .013, .106 | .013* |
|  | *N* patients | 233 |  |  |
|  | *N* observations | 739 |  |  |
| Diastolic BP ^a^ | Dose | .006 | -.085, .099 | .897 |
|  | Follow-up duration | .088 | .055, .120 | <.001*** |
|  | *N* patients | 233 |  |  |
|  | *N* observations | 739 |  |  |
| Glucose ^b^ | Dose | -.015 | -.031, .002 | .086 |
|  | Follow-up duration | -.018 | -.030, -.005 | .008** |
|  | *N* patients | 139 |  |  |
|  | *N* observations | 228 |  |  |
| Triglycerides ^c^ | Dose | .019 | .002, .023 | .023* |
|  | Follow-up duration | .005 | .005, .014 | <.001*** |
|  | *N* patients | 161 |  |  |
|  | *N* observations | 296 |  |  |
| Total Choles-terol ^c^ | Dose | .017 | .008, .025 | <.001*** |
|  | Follow-up duration | .001 | -.003, .005 | .549 |
|  | *N* patients | 161 |  |  |
|  | *N* observations | 294 |  |  |
| HDL-Cholesterol ^c^ | Dose | .002 | -.005, .003 | .585 |
|  | Follow-up duration | -.001 | -.003, .000 | .064 |
|  | *N* patients | 161 |  |  |
|  | *N* observations | 296 |  |  |
| LDL-Cholesterol ^c^ | Dose | .013 | .005, .021 | .001** |
|  | Follow-up duration | -.002 | -.006, .001 | .222 |
|  | *N* patients | 161 |  |  |
|  | *N* observations | 296 |  |  |
| nonHDL- Cholesterol ^c^ | Dose | .018 | .009, .026 | <.001*** |
|  | Follow-up duration | .001 | -.003, .005 | .575 |
|  | *N* patients | 161 |  |  |
|  | *N* observations | 296 |  |  |

*Note:* Abbreviations: APs: Antipsychotics; CI: Confidence interval; BMI: Body mass index; HDL: High-density lipoprotein; LDL: Low-density lipoprotein; BP: Blood pressure

^#^ Mixed effects models using olanzapine equivalent dose as the independent variables, cardiometabolic indices as the dependent variables; For BMI, waist circumference, systolic BP, and diastolic BP, follow-up was conducted for up to 5 years. Glucose, TG, TC, HDL-C, LDL-C, and nonHDL-C were followed up for up to 4 years.

* P<0.05, ** P<0.01, *** P<0.001

^a.^ Adjusted for follow-up duration, age, sex, smoking status, APs metabolic risk group, switch APs between different metabolic risk groups during follow-up, APs polypharmacy, concomitant use of other medications (antidepressants, benzodiazepines, mood stabilizers, lowering glucose, lowering fat, anti-hypertensive drugs) and baseline BMI, waist circumference, systolic BP, diastolic BP respectively;

^b.^ Adjusted for follow-up duration, age, sex, smoking status, APs metabolic risk group, switch different metabolic risk medications during follow-up, concomitant use of medications (antidepressants, benzodiazepines, mood stabilizers, lowering glucose, antihypertensive drugs), fasting status and baseline glucose.

^c.^ Adjusted for follow-up duration, age, sex, smoking status, APs metabolic risk group, switch different metabolic risk medications during follow-up, concomitant use of medications (antidepressants, benzodiazepines, mood stabilizers, lowering glucose, antihypertensive drugs), baseline outcome values (baseline TG, TC, HDL-C, LDL-C, nonHDL-C respectively).

# **Sensitivity analyses: including only olanzapine users**

Table S19. Effect of olanzapine dose reduction from baseline magnitude on changes in movement disorders over time.

| Outcomes | Variables | Estimates | 95% CI | P-value |
| --- | --- | --- | --- | --- |
| Parkinsonism ^a^ | Reduced dose | -.003 | -.015, .008 | .556 |
|  | Follow-up duration | .002 | -.002, .006 | .268 |
|  | *N* patients | 78 |  |  |
|  | *N* observations | 181 |  |  |
| Dyskinesia ^a^ | Reduced dose | -.008 | -.013, -.003 | .002* |
|  | Follow-up duration | -.000 | -.003, .002 | .781 |
|  | *N* patients | 79 |  |  |
|  | *N* observations | 185 |  |  |
| Akathisia ^a^ | Reduced dose | -.001 | -.013, .011 | .912 |
|  | Follow-up duration | .004 | -.001, .009 | .101 |
|  | *N* patients | 77 |  |  |
|  | *N* observations | 181 |  |  |
| Dystonia ^a^ | Reduced Dose | .000 | -.013, .013 | .951 |
|  | Follow-up duration | .011 | .005, .017 | <.001*** |
|  | *N* patients | 80 |  |  |
|  | *N* observations | 191 |  |  |

*Note*: APs: Antipsychotics; CI: Confidence interval

* P<0.05, ** P<0.01, *** P<0.001

a. Adjusted for follow-up duration, age, sex, smoking status, APs D2R affinity groups, switch APs between different D2R affinity groups during follow-up, APs polypharmacy, concomitant use of other medications (antidepressants, benzodiazepines, mood stabilizers, anti-movement disorders medications) and baseline outcome values (baseline mean scores of parkinsonism, dyskinesia, akathisia and global dystonia score respectively)

Table S20. Effect of olanzapine dose reduction from baseline magnitude on changes in cardiometabolic indices over time.

| Outcomes^#^ | Variables | Estimates | 95% CI | P-value |
| --- | --- | --- | --- | --- |
| BMI ^a^ | Reduced dose | -.010 | -.048 .030 | .614 |
|  | Follow-up duration | .006 | -.005, .017 | .310 |
|  | *N* patients | 99 |  |  |
|  | *N* observations | 336 |  |  |
| Waist circumference ^b^ | Reduced dose | -.219 | -.421, -.007 | .047* |
|  | Follow-up duration | .094 | .037, .151 | .002** |
|  | *N* patients | 77 |  |  |
|  | *N* observations | 183 |  |  |
| Systolic BP ^c^ | Reduced dose | .062 | -.229, .342 | .686 |
|  | Follow-up duration | .081 | -.009, .166 | .078 |
|  | *N* patients | 75 |  |  |
|  | *N* observations | 185 |  |  |
| Diastolic BP ^c^ | Reduced Dose | .030 | -.196, .254 | .805 |
|  | Follow-up duration | .100 | .035, .161 | .003** |
|  | *N* patients | 75 |  |  |
|  | *N* observations | 185 |  |  |
| Glucose ^d^ | Reduced Dose | .002 | -.019, .024 | .861 |
|  | Follow-up duration | .004 | -.012, .020 | .670 |
|  | *N* patients | 43 |  |  |
|  | *N* observations | 52 |  |  |
| Triglycerides ^e^ | Reduced Dose | .009 | -.011, .028 | .418 |
|  | Follow-up duration | .002 | -.007, .012 | .668 |
|  | *N* patients | 46 |  |  |
|  | *N* observations | 60 |  |  |
| Total Choles-terol ^e^ | Reduced Dose | -.020 | -.043, .004 | .137 |
|  | Follow-up duration | -.002 | -.015, .010 | .807 |
|  | *N* patients | 45 |  |  |
|  | *N* observations | 58 |  |  |
| HDL-Cholesterol ^e^ | Reduced Dose | -.000 | -.008, .008 | .967 |
|  | Follow-up duration | .000 | -.004, .003 | .990 |
|  | *N* patients | 46 |  |  |
|  | *N* observations | 60 |  |  |
| LDL-Cholesterol ^e^ | Reduced Dose | -.016 | -.037, .006 | .201 |
|  | Follow-up duration | -.009 | -.020, .001 | .141 |
|  | *N* patients | 46 |  |  |
|  | *N* observations | 60 |  |  |
| nonHDL- Cholesterol ^e^ | Reduced Dose | -.021 | -.044, .002 | .116 |
|  | Follow-up duration | -.005 | -.017, .006 | .434 |
|  | *N* patients | 46 |  |  |
|  | *N* observations | 60 |  |  |

*Note:* Abbreviations: APs: Antipsychotics; CI: Confidence interval; BMI: Body mass index; HDL: High-density lipoprotein; LDL: Low-density lipoprotein; BP: Blood pressure

* P<0.05, ** P<0.01, *** P<0.001

^#^ For BMI, waist circumference, systolic BP, and diastolic BP, follow-up was conducted for up to 5 years. Glucose, TG, TC, HDL-C, LDL-C, and nonHDL-C were followed up for up to 4 years.

a. Adjusted for follow-up duration, age, sex, smoking status, AP metabolic risk group, switch AP between different metabolic risk groups during follow-up, AP polypharmacy, concomitant use of other medications (antidepressants, benzodiazepines, mood stabilizers, lowering glucose, anti-hypertensive drugs), and baseline BMI.

b. Adjusted for follow-up duration, age, sex, smoking status, AP metabolic risk group, switch AP between different metabolic risk groups during follow-up, AP polypharmacy, concomitant use of other medications (antidepressants, benzodiazepines, mood stabilizers, anti-hypertensive drugs), and baseline waist circumference.

c. Adjusted for follow-up duration, age, sex, smoking status, APs metabolic risk group, switch APs between different metabolic risk groups during follow-up, APs polypharmacy, concomitant use of other medications (antidepressants, benzodiazepines, mood stabilizers, anti-hypertensive drugs), and baseline systolic and diastolic blood pressure respectively.

d. Adjusted for follow-up duration, age, sex, smoking status, APs metabolic risk group, switch APs between different metabolic risk groups during follow-up, APs polypharmacy, baseline outcome values, fasting, concomitant use of other medications, (antidepressants, benzodiazepines, mood stabilizers, anti-hypertensive drugs), and baseline glucose.

e. Adjusted for follow-up duration, age, sex, smoking status, APs metabolic risk group, switch APs between different metabolic risk groups during follow-up, APs polypharmacy, concomitant use of other medications, (antidepressants, benzodiazepines, mood stabilizers), baseline outcome values (baseline TG, TC, HDL-C, LDL-C, nonHDL-C respectively).

Table S21. Effect of time-lagged olanzapine dose reduction magnitude on changes in movement disorders over time.

| Outcomes | Variables | Estimates | 95% CI | P-value |
| --- | --- | --- | --- | --- |
| Parkinsonism ^a^ | Reduced dose | -.004 | -.018, .010 | .607 |
|  | Follow-up duration | .005 | -.001, .011 | .110 |
|  | *N* patients | 72 |  |  |
|  | *N* observations | 150 |  |  |
| Dyskinesia ^a^ | Reduced dose | -.009 | -.018, .000 | .063 |
|  | Follow-up duration | .000 | -.004, .004 | .957 |
|  | *N* patients | 74 |  |  |
|  | *N* observations | 154 |  |  |
| Akathisia ^a^ | Reduced dose | .001 | -.017, .019 | .932 |
|  | Follow-up duration | .002 | -.005, .010 | .558 |
|  | *N* patients | 73 |  |  |
|  | *N* observations | 149 |  |  |
| Dystonia ^a^ | Reduced Dose | .004 | -.015, .022 | .721 |
|  | Follow-up duration | .013 | .005, .020 | .002** |
|  | *N* patients | 76 |  |  |
|  | *N* observations | 163 |  |  |

*Note*: APs: Antipsychotics; CI: Confidence interval

* P<0.05, ** P<0.01, *** P<0.001

a. Adjusted for follow-up duration, age, sex, smoking status, APs D2R affinity groups, switch APs between different D2R affinity groups during follow-up, APs polypharmacy, concomitant use of other medications (antidepressants, benzodiazepines, mood stabilizers, anti-movement disorders medications) and baseline outcome values (baseline mean scores of parkinsonism, dyskinesia, akathisia and global dystonia score respectively)

Table S22. Effect of time-lagged olanzapine dose reduction magnitude on changes in cardiometabolic indices over time.

| Outcomes^#^ | Variables | Estimates | 95% CI | P-value |
| --- | --- | --- | --- | --- |
| BMI ^a^ | Reduced dose | -.002 | -.037, .034 | .933 |
|  | Follow-up duration | .002 | -.009, .013 | .731 |
|  | *N* patients | 97 |  |  |
|  | *N* observations | 306 |  |  |
| Waist circumference ^b^ | Reduced dose | -.137 | -.351, .076 | .231 |
|  | Follow-up duration | .046 | -.041, .132 | .324 |
|  | *N* patients | 58 |  |  |
|  | *N* observations | 130 |  |  |
| Systolic BP ^c^ | Reduced dose | .043 | -.340, .427 | .832 |
|  | Follow-up duration | -.051 | -.201, .099 | .525 |
|  | *N* patients | 60 |  |  |
|  | *N* observations | 131 |  |  |
| Diastolic BP ^c^ | Reduced Dose | -.075 | -.373, .223 | .639 |
|  | Follow-up duration | -.007 | -.123, .109 | .912 |
|  | *N* patients | 60 |  |  |
|  | *N* observations | 131 |  |  |
| Glucose ^d^ | Reduced Dose | .010 | -.039, .060 | .726 |
|  | Follow-up duration | .019 | -.003, .042 | .160 |
|  | *N* patients | 37 |  |  |
|  | *N* observations | 45 |  |  |
| Triglycerides ^e^ | Reduced Dose | .012 | -.034, .059 | .644 |
|  | Follow-up duration | .003 | -.010, .015 | .646 |
|  | *N* patients | 41 |  |  |
|  | *N* observations | 52 |  |  |
| Total Choles-terol ^e^ | Reduced Dose | -.004 | -.057, .050 | .909 |
|  | Follow-up duration | .010 | -.005, .025 | .264 |
|  | *N* patients | 40 |  |  |
|  | *N* observations | 51 |  |  |
| HDL-Cholesterol ^e^ | Reduced Dose | -.004 | -.017, .009 | .601 |
|  | Follow-up duration | .000 | -.003, .004 | .840 |
|  | *N* patients | 41 |  |  |
|  | *N* observations | 52 |  |  |
| LDL-Cholesterol ^e^ | Reduced Dose | -.003 | -.052, .045 | .902 |
|  | Follow-up duration | -.000 | -.013, .013 | .997 |
|  | *N* patients | 41 |  |  |
|  | *N* observations | 52 |  |  |
| nonHDL- Cholesterol ^e^ | Reduced Dose | .002 | -.051, .054 | .959 |
|  | Follow-up duration | .005 | -.009, .019 | .540 |
|  | *N* patients | 41 |  |  |
|  | *N* observations | 52 |  |  |

*Note:* Abbreviations: APs: Antipsychotics; CI: Confidence interval; BMI: Body mass index; HDL: High-density lipoprotein; LDL: Low-density lipoprotein; BP: Blood pressure

* P<0.05, ** P<0.01, *** P<0.001

^#^ For BMI, waist circumference, systolic BP, and diastolic BP, follow-up was conducted for up to 5 years. Glucose, TG, TC, HDL-C, LDL-C, and nonHDL-C were followed up for up to 4 years.

a. Adjusted for follow-up duration, age, sex, smoking status, APs metabolic risk group, switch APs between different metabolic risk groups during follow-up, APs polypharmacy, concomitant use of other medications (antidepressants, benzodiazepines, mood stabilizers, lowering glucose, anti-hypertensive drugs), and baseline BMI.

b. Adjusted for follow-up duration, age, sex, smoking status, APs metabolic risk group, switch APs between different metabolic risk groups during follow-up, APs polypharmacy, concomitant use of other medications (antidepressants, benzodiazepines, mood stabilizers, anti-hypertensive drugs), and baseline waist circumference.

c. Adjusted for follow-up duration, age, sex, smoking status, APs metabolic risk group, switch APs between different metabolic risk groups during follow-up, APs polypharmacy, concomitant use of other medications (antidepressants, benzodiazepines, mood stabilizers, anti-hypertensive drugs), and baseline systolic and diastolic blood pressure respectively.

d. Adjusted for follow-up duration, age, sex, smoking status, APs metabolic risk group, switch APs between different metabolic risk groups during follow-up, APs polypharmacy, baseline outcome values, fasting, concomitant use of other medications, (antidepressants, benzodiazepines, mood stabilizers, anti-hypertensive drugs), and baseline glucose.

e. Adjusted for follow-up duration, age, sex, smoking status, APs metabolic risk group, switch APs between different metabolic risk groups during follow-up, APs polypharmacy, concomitant use of other medications, (antidepressants, benzodiazepines, mood stabilizers), baseline outcome values (baseline TG, TC, HDL-C, LDL-C, nonHDL-C respectively).

Table S23. Effect of olanzapine dose on movement disorders over time.

| Outcomes^#^ |  | Variables | Estimates^##^ | 95% CI | P-value |
| --- | --- | --- | --- | --- | --- |
| Parkinsonism ^a^ | *Part 1* | Dose | 0.871 | 0.813, 0.933 | <.001*** |
|  |  | Follow-up duration | 0.994 | 0.971, 1.018 | .637 |
|  | *Part 2* | Dose | 0.012 | 0.000, 0.022 | .049* |
|  |  | Follow-up duration | 0.002 | -0.002, 0.007 | .316 |
|  |  | *N* patients | 93 |  |  |
|  |  | *N* observations | 272 |  |  |
| Dyskinesia ^a^ | *Part 1* | Dose | 0.930 | 0.873, 0.991 | .024* |
|  |  | Follow-up duration | 1.019 | 0.986, 1.053 | .257 |
|  | *Part 2* | Dose | 0.008 | -0.004, 0.021 | .243 |
|  |  | Follow-up duration | 0.003 | -0.004, 0.010 | .431 |
|  |  | *N* patients | 95 |  |  |
|  |  | *N* observations | 278 |  |  |
| Akathisia ^a^ | *Part 1* | Dose | 0.985 | 0.933, 1.039 | .574 |
|  |  | Follow-up duration | 0.994 | 0.972, 1.017 | .628 |
|  | *Part 2* | Dose | 0.015 | 0.000, 0.029 | .069 |
|  |  | Follow-up duration | 0.009 | 0.003, 0.016 | .007** |
|  |  | *N* patients | 92 |  |  |
|  |  | *N* observations | 271 |  |  |
| Dystonia ^a^ | *Part 1* | Dose | 0.945 | 0.877, 1.018 | .135 |
|  |  | Follow-up duration | 0.955 | 0.927, 0.985 | .003** |
|  | *Part 2* | Dose | 0.046 | -0.000 0.093 | .114 |
|  |  | Follow-up duration | 0.030 | 0.012, 0.047 | .010** |
|  |  | *N* patients | 96 |  |  |
|  |  | *N* observations | 285 |  |  |

*Note*: APs: Antipsychotics; CI: Confidence interval

* P<0.05, ** P<0.01, *** P<0.001

^#^ Two-part mixed effects models: Part 1: Generalized linear mixed binary models using olanzapine dose as the independent variable, and zero or non-zero mean scores of movement disorder scales (binary, 1 = zero mean scores, 0 = non-zero mean scores) as the dependent variables, Part 2: Linear mixed models, using olanzapine dose as the independent variable, and non-zero mean scores of movement disorder scales (continuous) as the dependent variables

^##^ In the part 1 models, the estimates are odds ratios; In the part 2 models, the estimates are β coefficients.

a. Adjusted for follow-up duration, age, sex, smoking status, APs D2R affinity groups, switch APs between different D2R affinity groups during follow-up, APs polypharmacy, concomitant use of other medications (antidepressants, benzodiazepines, mood stabilizers) and baseline outcome values (baseline mean scores of parkinsonism, dyskinesia, akathisia and baseline global dystonia score respectively)

Table S24. Effect of olanzapine dose on cardiometabolic indices over time.

| Outcomes^#^ | Variables | Estimates | 95% CI | P-value |
| --- | --- | --- | --- | --- |
| BMI ^a^ | Dose | .023 | -.007, .055 | .151 |
|  | Follow-up duration | .009 | -.001, .019 | .089 |
|  | *N* patients | 106 |  |  |
|  | *N* observations | 440 |  |  |
| Waist circumference ^a^ | Dose | .233 | .093, .373 | .002** |
|  | Follow-up duration | .087 | .040, .135 | <.001*** |
|  | *N* patients | 86 |  |  |
|  | *N* observations | 267 |  |  |
| Systolic BP ^a^ | Dose | -.120 | -.329, .088 | .275 |
|  | Follow-up duration | .067 | -.010, .141 | .087 |
|  | *N* patients | 84 |  |  |
|  | *N* observations | 267 |  |  |
| Diastolic BP ^a^ | Dose | .058 | -.101, .221 | .497 |
|  | Follow-up duration | .093 | .036, .147 | .001** |
|  | *N* patients | 84 |  |  |
|  | *N* observations | 267 |  |  |
| Glucose ^b^ | Dose | .012 | -.001, .025 | .104 |
|  | Follow-up duration | -.007 | -.016, .003 | .230 |
|  | *N* patients | 58 |  |  |
|  | *N* observations | 105 |  |  |
| Triglycerides ^c^ | Dose | .003 | -.011, .017 | .671 |
|  | Follow-up duration | -.003 | -.010, .005 | .516 |
|  | *N* patients | 65 |  |  |
|  | *N* observations | 124 |  |  |
| Total Choles-terol ^c^ | Dose | .014 | .001, .028 | .047* |
|  | Follow-up duration | -.010 | -.017, -.002 | .025* |
|  | *N* patients | 64 |  |  |
|  | *N* observations | 121 |  |  |
| HDL-Cholesterol ^c^ | Dose | -.002 | -.006, .002 | .419 |
|  | Follow-up duration | -.002 | -.004, .000 | .109 |
|  | *N* patients | 65 |  |  |
|  | *N* observations | 124 |  |  |
| LDL-Cholesterol ^c^ | Dose | .012 | .001, .023 | .049* |
|  | Follow-up duration | -.012 | -.018, -.006 | <.001*** |
|  | *N* patients | 65 |  |  |
|  | *N* observations | 124 |  |  |
| nonHDL- Cholesterol ^c^ | Dose | .015 | .001, .028 | .044* |
|  | Follow-up duration | -.011 | -.019, -.004 | .003** |
|  | *N* patients | 65 |  |  |
|  | *N* observations | 124 |  |  |

*Note:* Abbreviations: CI: Confidence interval; BMI: Body mass index; HDL: High-density lipoprotein; LDL: Low-density lipoprotein; BP: Blood pressure

* P<0.05, ** P<0.01, *** P<0.001

# For BMI, waist circumference, systolic BP, and diastolic BP, follow-up was conducted for up to 5 years. Glucose, triglycerides, total cholesterol, HDL-cholesterol, LDL-cholesterol, and nonHDL-cholesterol were followed up for up to 4 years.

^a.^ Adjusted for follow-up duration, age, sex, smoking status, APs metabolic risk group, switch APs between different metabolic risk groups during follow-up, APs polypharmacy, concomitant use of other medications (antidepressants, benzodiazepines, mood stabilizers, lowering glucose, anti-hypertensive drugs) and baseline BMI, waist circumference, systolic BP, diastolic BP respectively;

^b.^ Adjusted for follow-up duration, age, sex, smoking status, APs metabolic risk group, switch different metabolic risk medications during follow-up, concomitant use of medications (antidepressants, benzodiazepines, mood stabilizers, antihypertensive drugs), fasting, baseline glucose.

^c.^ Adjusted for follow-up duration, age, sex, smoking status, APs metabolic risk group, switch different metabolic risk medications during follow-up, concomitant use of medications (antidepressants, benzodiazepines, mood stabilizers, antihypertensive drugs), baseline triglycerides, total cholesterol, HDL-cholesterol, LDL-cholesterol, and nonHDL-cholesterol respectively.

# **Figure**

Figure 1. Forest plot illustrating the effect of each predictor on the change in mean dyskinesia scores on the St. Hans Rating Scale from baseline.


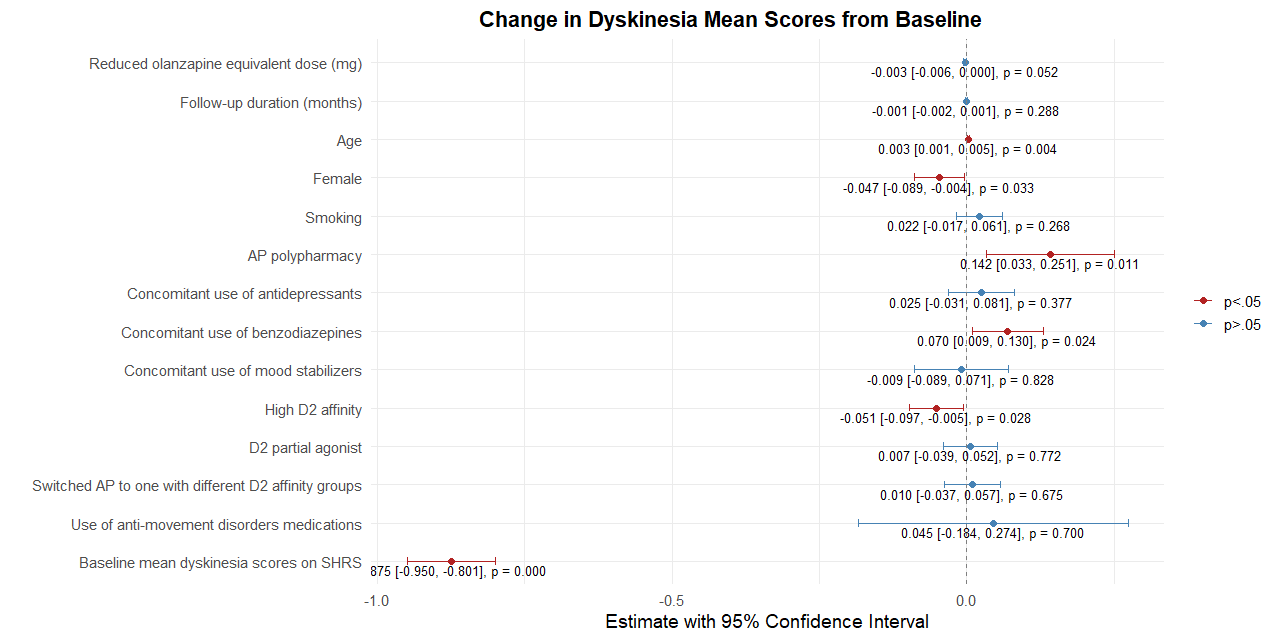


# **Reference**

1. Pieters LE, Deenik J, Tenback DE, van Oort J, van Harten PN. Exploring the Relationship Between Movement Disorders and Physical Activity in Patients With Schizophrenia: An Actigraphy Study. Schizophr Bull. 2021; 47(4): 906-14.

2. Gerlach J, Korsgaard S, Clemmesen P, Lauersen AM, Magelund G, Noring U, et al. The St. Hans Rating Scale for extrapyramidal syndromes: reliability and validity. Acta Psychiatr Scand. 1993; 87(4): 244-52.

3. Barnes TR. A rating scale for drug-induced akathisia. Br J Psychiatry. 1989; 154: 672-6.

4. Must A, Spadano J, Coakley EH, Field AE, Colditz G, Dietz WH. The disease burden associated with overweight and obesity. JAMA. 1999; 282(16): 1523-9.

5. Alberti KG, Zimmet P, Shaw J. Metabolic syndrome--a new world-wide definition. A Consensus Statement from the International Diabetes Federation. Diabet Med. 2006; 23(5): 469-80.

6. LaRosa JC. At what levels of total low- or high-density lipoprotein cholesterol should diet/drug therapy be initiated? United States guidelines. Am J Cardiol. 1990; 65(12): 7F-10F.

7. Mach F, Baigent C, Catapano AL, Koskinas KC, Casula M, Badimon L, et al. 2019 ESC/EAS Guidelines for the management of dyslipidaemias: lipid modification to reduce cardiovascular risk. Eur Heart J. 2020; 41(1): 111-88.

8. Raja V, Aguiar C, Alsayed N, Chibber YS, ElBadawi H, Ezhov M, et al. Non-HDL-cholesterol in dyslipidemia: Review of the state-of-the-art literature and outlook. Atherosclerosis. 2023; 383: 117312.

9. Liu L, Shih Y-CT, Strawderman RL, Zhang D, Johnson BA, Chai H. Statistical Analysis of Zero-Inflated Nonnegative Continuous Data A Review. Statistical Science. 2019; 34(2): 253-79.
